# Publisher Correction: Conversion of triphenylphosphine oxide to organophosphorus via selective cleavage of C-P, O-P, and C-H bonds with sodium

**DOI:** 10.1038/s42004-020-0266-5

**Published:** 2020-02-06

**Authors:** Jian-Qiu Zhang, Jingjing Ye, Tianzeng Huang, Hiroyuki Shinohara, Hiroyoshi Fujino, Li-Biao Han

**Affiliations:** 1 National Institute of Advanced Industrial Science and Technology (AIST), Tsukuba, Ibaraki 305-8565 Japan; 2 Division of Chemistry, Faculty of Pure and Applied Sciences, University of Tsukuba, Tsukuba, Ibaraki 305-8571 Japan; 3 Katayama Chemical Industries Co., Ltd., 26-22, 3-Chome, Higasinaniwa-cho, Amagasaki, Hyogo 660-0892 Japan

**Keywords:** Sustainability, Synthetic chemistry methodology

Correction to: *Communications Chemistry* 10.1038/s42004-019-0249-6, published online 3 January 2020.

The original version of this Article contained an error in reference 2. The title in reference 2 was incorrectly written as ‘The Staudinger ligation-a.pngt to chemical biology’. This error has been corrected in the HTML and PDF versions of the Article.

