# Supplementary material for: Conversion of triphenylphosphine oxide to organophosphorus via selective cleavage of C-P, O-P, and C-H bonds with sodium
Source: Commun Chem. 2020 Jan 3;3:1. doi: 10.1038/s42004-019-0249-6 (PMC9812262; doi:10.1038/s42004-019-0249-6)
Supplement: Supplementary file 1 — Supplementary Information [file 42004_2019_249_MOESM1_ESM.pdf]

Supplementary Information

Supplementary Methods
General information

All reactions were carried out in oven dried Schlenk tubes under argon atmosphere. All materials were purchased and used without further purification. The sodium we used was dispersed in paraffin oil with an average particle size smaller than 10 μm and a concentration of 10 mol/L. ^{1}H NMR spectra were recorded on JEOL JNM-ECS400 (400 MHz) FT NMR in $CDCl_3$ with Me_4Si as an internal standard. ^{13}C NMR spectra were taken on JEOL JNM-ECS400 (100 MHz) FT NMR system in $CDCl_3$. ^{31}P NMR spectra were taken on JEOL JNM-ECX400 (162 MHz) FT NMR system in $CDCl_3$ with 85% H_3PO_4 solution as an external standard. MS (ESI) data were obtained on SHIMADZU GC-MS 2010 plus. HPLC (recycle GPC) method for isolation was performed on JAPAN ANALYTICAL INDUSTRY LC-908. The HPLC was a recycling preparative HPLC (Gel permeation chromatography) with two columns (20 mm I.D. -600 mm L; JAIGEL-1H and JAIGEL-2H). All the products were purified by the same conditions: $CHCl_3$ as eluent, flow rate: 4.0 mL/min, and the spectra was recorded at a moving paper in the speed of 60 mm/h. Melting points were measured on OptiMelt by SRS.

General procedures for selective transformation of Ph3P(O) to 2, 3, 4

General method A for selective transformation of $Ph_3P(O)$ to sodium diphenylphosphinite 2 and its derivatives

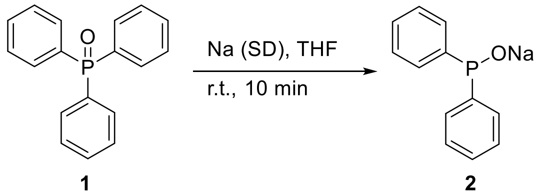

Supplementary Figure 1. Conversion of $Ph_3P(O)$ to **2**

Under the argon atmosphere (1 atm), 1.0 mmol triphenylphosphine oxide **1** (278 mg) was dissolved in 5 mL solvent (THF or 1,4-dioxane), and then 2.5 mmol SD (Sodium Dispersed, 0.25 mL) was added in dropwise by syringe within 10 seconds. The mixture was stirred under room temperature for 10 minutes. After reaction, excessive solid Na was separated from the solution by centrifugation, product *sodium diphenylphosphinite* **2** was determined by ^{31}P NMR with a signal at 91 ppm in quantitative yield.

(1) For the synthesis of $Ph_2P(O)H$:

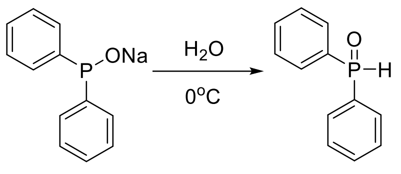

2 **2-1**

Supplementary Figure 2. Conversion of $Ph_2P(ONa)$ **2** to $Ph_2P(O)H$

According to general method A, sodium diphenylphosphinite **2** was obtained in 1,4-dioxane instead of THF for higher yield. Under argon (1 atm), 1.0 mmol Ph_2PONa (dioxane solution) was quenched by 2.0 mL saturated aqueous NH_4Cl solution and extracted with EtOAc (3 mL×3), the combined organic layer was dried over anhydrous Na_2SO_4, then filtered and evaporated. The crude product was washed by Hexane (5 mL×3) via a silica column to remove the oil derived from SD and eluent with EtOAc to give crude products. And recrystallization (5.0 mL Hexane + 2 mL $CHCl_3$ at -20°C) afforded analytically pure $Ph_2P(O)H$ **2-1** in quantitative yield.

(2) For the synthesis of $Ph_2P(O)C(O)Mes$：

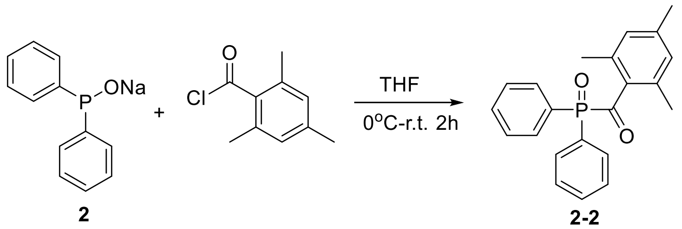

2 **2-2**

Supplementary Figure 3. Conversion of $Ph_2P(ONa)$ **2** to TPO

Under argon (1 atm), 1.0 mmol Ph_2PONa (THF solution) was added dropwise to

1.5 mmol MesC(O)Cl dissolved in 5.0 mL THF at 0°C, after stirring for 2 hours at room temperature, the mixture was quenched by 5.0 mL saturated aqueous NH_4Cl solution and extracted with EtOAc (5 mL×3), the combined organic layer was dried over anhydrous Na_2SO_4, then filtered and evaporated. The residues were passed through a silica chromatographic column (particle size 37-54 µm) using ethyl acetate/petroleum ether (1:4) as an eluent to afford analytically pure **2-2** product in 56% isolated yield.

(3) For the synthesis of $Ph_2P(O)R$:

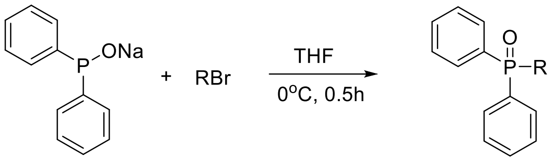

Supplementary Figure 4. Conversion of $Ph_2P(ONa)$ **2** to diphenylalkylphosphine oxides

Under argon (1 atm), 1.2 mmol electrophilic reagents RBr was added dropwise to 1.0 mmol Ph_2PONa (THF solution) at 0°C. After stirring for 0.5 h, the mixture was washed by 5.0 mL saturated aqueous NH_4Cl solution, and then extracted with EtOAc (5 mL×3), the combined organic layer was dried over anhydrous Na_2SO_4, then filtered and evaporated. The residues were diluted in $CHCl_3$ and isolated by HPLC (polystyrene-based column, $CHCl_3$ as eluent, flow rate = 4.0 mL/min) to afford analytically pure **2-3** products.

General method B for selective transformation of $Ph_3P(O)$ to sodium 5H-benzo[b]phosphindol-5-olate 3 and its derivatives

Under the argon (1 atm), to a solution of 2.0 mmol sodium dispersion (0.2 mL) in 2 mL of hexane was added 1.1 mmol PhCl (112 µL) and the mixture was stirred at room temperature for 1 h until the color of mixture turned deep purple. And then the solution of 0.9 mmol $Ph_3P(O)$ (250 mg) dissolved in 2 mL of THF was added to the above PhNa/hexane solution *via* syringe within 10 seconds. The reaction proceeded under room temperature for overnight. The selective product *sodium 5H-benzo[b]phosphindol-5-olate* **3** was determined by ^{31}P-NMR with a signal at 101

ppm. And then the mixture of **3** was quenched with 1.5 mmol RBr at 0°C and stirred for 0.5 h, washed by saturated aqueous NH_4Cl solution, extracted with EtOAc (5 mL×3), the combined organic layer was dried over anhydrous Na_2SO_4, then filtered and evaporated. The residues were diluted in $CHCl_3$ and isolated by HPLC (polystyrene-based column, $CHCl_3$ as eluent, flow rate = 4.0 mL/min) to afford analytically pure **3'** products.

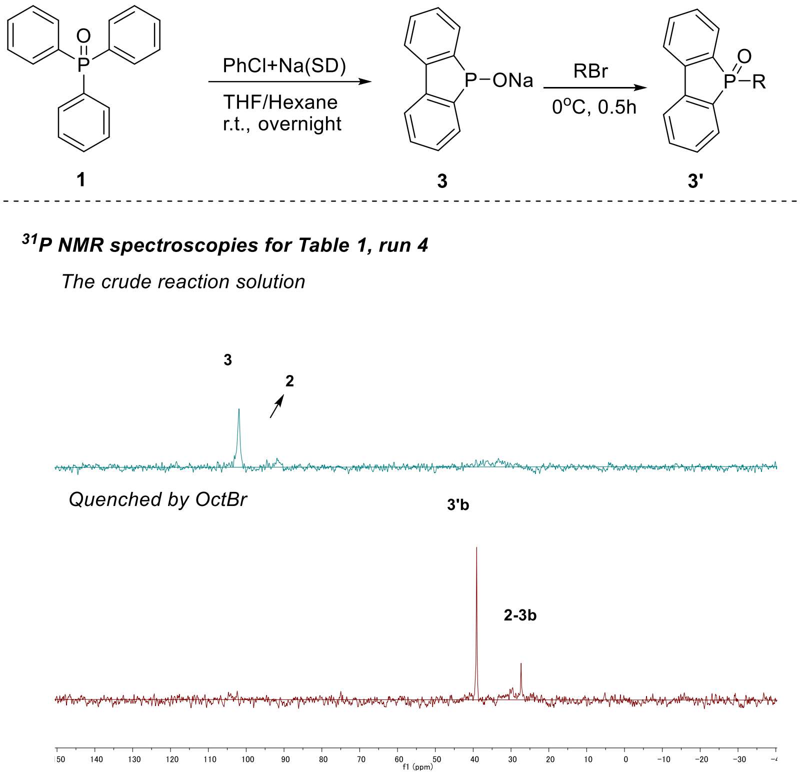

Supplementary Figure 5. Conversion of $Ph_3P(O)$ to *sodium 5H-benzo[b]phosphindol-5-olate* **3** and quenching with alkyl bromides

General method C for selective transformation of $Ph_3P(O)$ to sodium benzo[b]phosphindol-5-ide 4 and its derivative

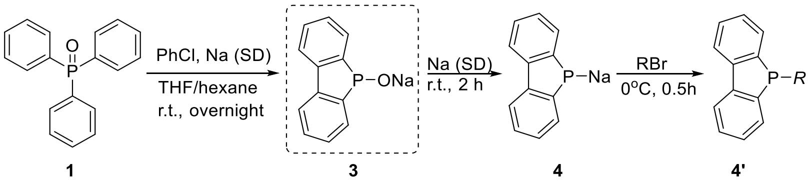

Supplementary Figure 6. Conversion of $Ph_3P(O)$ to *sodium benzo[b]phosphindol-5-ide* **4** and

quenching with alkyl bromides

Under the argon atmosphere (1 atm), to a solution of 2.0 mmol sodium dispersion (0.2 mL) in 2 mL of hexane was added 1.1 mmol PhCl (112 µL) and the mixture was stirred at room temperature for 1h until the color of mixture turned deep purple. And then the solution of 0.9 mmol $Ph_3P(O)$ (250 mg) dissolved in 2 mL of THF was added to the above PhNa solution *via* syringe within 10 seconds. The reaction proceeded under room temperature for overnight affording the solution of **3**, and then 2.5 mmol sodium dispersion (0.25 mL) was added and the mixture was stirred at room temperature for 2 h. The selective product *sodium benzo[b]phosphindol-5-ide* **4** were determined by ^{31}P-NMR with a signal at 3.0 ppm and quenching with RBr affording 5-alkyl-5H-benzo[b]phosphindole **4'**. The residues were diluted in $CHCl_3$ and isolated by HPLC (polystyrene-based column, $CHCl_3$ as eluent, flow rate = 4.0 mL/min) to afford analytically pure **4'** products.

Scale-up synthesis of Ph2P(O)H

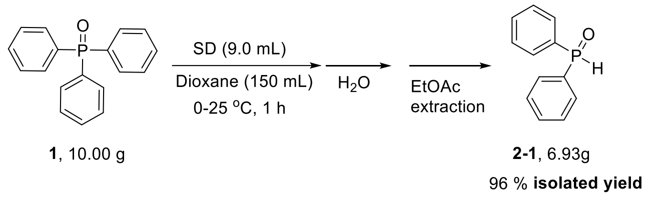

Supplementary Figure 7. 10g-scale up synthesis of $Ph_2P(O)H$ from $Ph_3P(O)$

Under the argon atmosphere (1 atm), 10.0 g triphenylphosphine oxide **1** (35.93 mmol) was dissolved in 150 mL 1,4-dioxane, and then 9.0 mL SD (Sodium Dispersed, 90 mmol) was added in dropwise at 0°C. After stirring for 1 hour under room temperature (**A**). The Ph_2PONa (dioxane solution) was quenched by 150 mL saturated aqueous NH_4Cl solution and extracted with EtOAc (100 mL×3) (**B**). The combined organic layer was dried over Na_2SO_4, then filtered and evaporated. The crude product was washed with hexane by column chromatography to remove the oil derived from SD, then elution with EtOAc to give the crude product. Evaporation of EtOAc and recrystallization (100 mL Hexane + 10 mL $HCCl_3$ at -20°C) afforded analytically pure $Ph_2P(O)H$ **2-1** (6.93 g) in 96 % isolated yield (**C**).

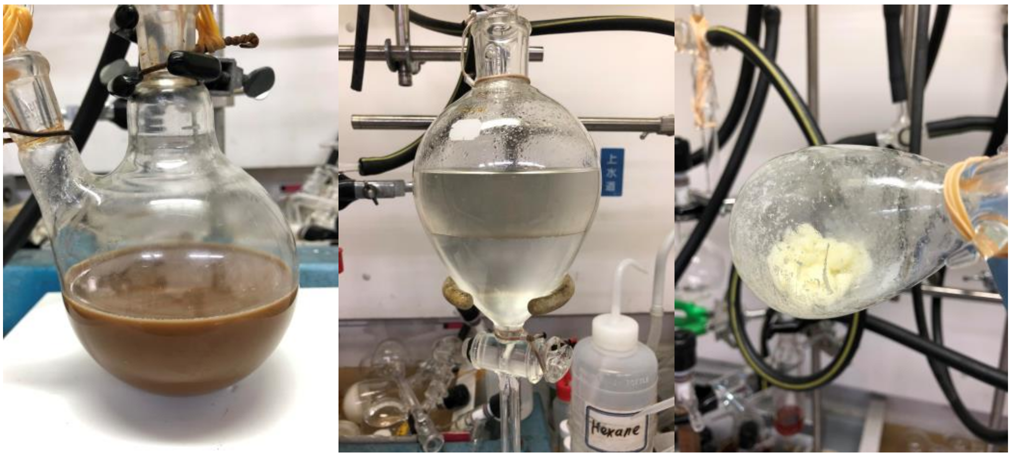

(A) **(B)** **(C)**

Supplementary Figure 8. (A)$Ph_3P(O)$+SD (dioxane), (B) Quenched with H_2O, (C) pure $Ph_2P(O)H$

MS (ESI) spectra copy of 2’, 3’ and 4’

Quenching the crude reaction mixture of **2**, **3**, **4** with *n*-BuBr afforded the three kinds of corresponding product **2’, 3’** and **4’**, which was detected by GCMS.

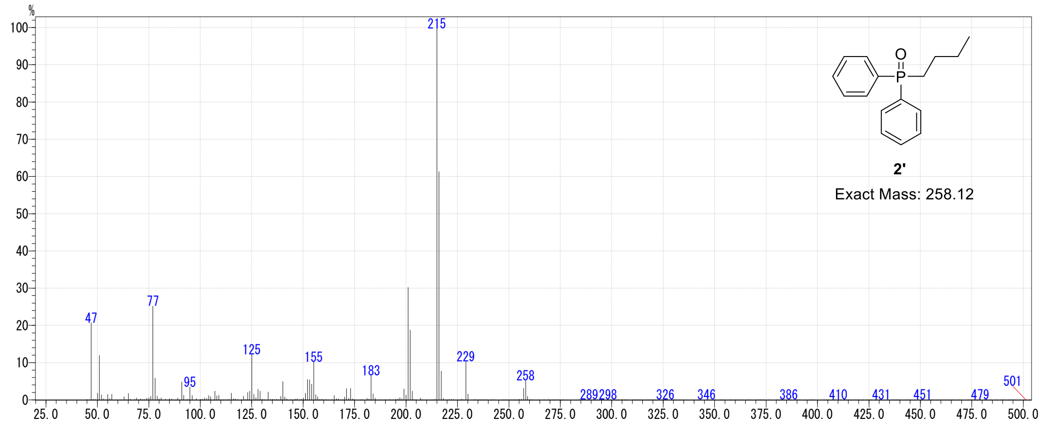

Supplementary Figure 9. MS (ESI) spectra copy of **2’**

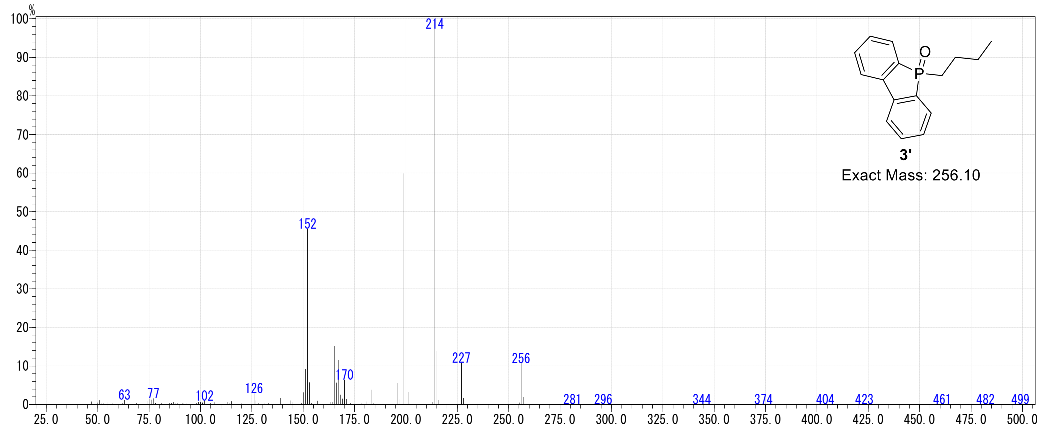

Supplementary Figure 10. MS (ESI) spectra copy of **3’**

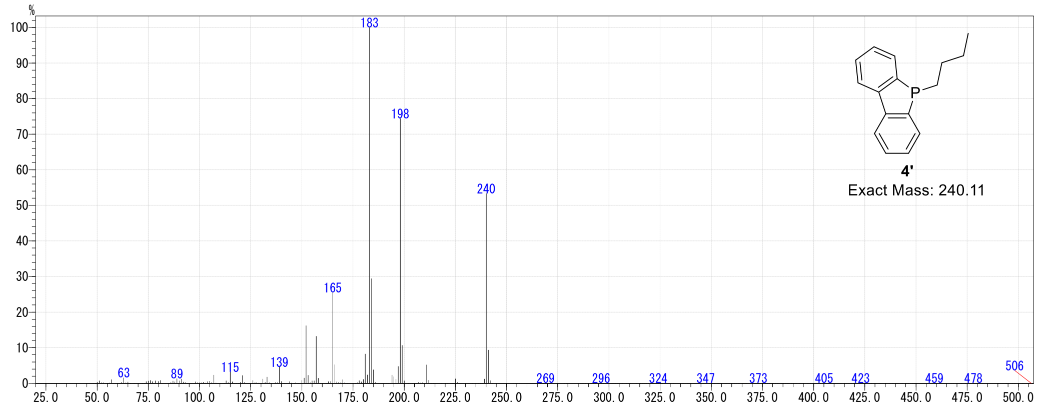

Supplementary Figure 11. MS (ESI) spectra copy of **4’**

Characterization and analytical data of products

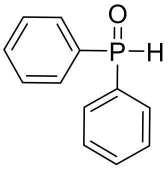

2-1

Diphenylphosphine oxide (2-1). According to the general procedure A-1, purification by recrystallization afforded **2-1** (197mg, 98% yield) as a pale yellow solid; ^{1}H NMR (400 MHz, $CDCl_3$): δ 8.63 (s, 0.5 H), 7.69–7.64 (m, 4H), 7.54–7.50 (m, 2H), 7.47–7.43 (m, 4.5H); ^{13}C NMR (100 MHz, $CDCl_3$): δ 132.62 (d, $J_{P\text{-}C}$ = 2.6 Hz), 131.55 (d, $J_{P\text{-}C}$ = 100.8 Hz), 130.76 (d, $J_{P\text{-}C}$ = 11.4 Hz), 128.96 (d, $J_{P\text{-}C}$ = 12.6 Hz); ^{31}P NMR (162 MHz, $CDCl_3$): δ 21.91. MS (ESI) m/z: ($[M]^+$) Calcd for $C_{12}H_{11}OP$ 202, found 202. Melting Point: 42.9-45.5 °C. This compound is known and in agreement with the literature data.[1]

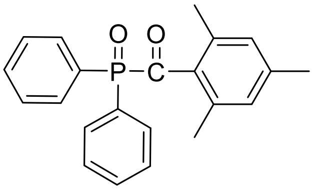

2-2

(diphenylphosphoryl)(mesityl)methanone (2-2). According to the general procedure A-2, purification by silica column chromatography afforded **2-2** (195 mg, 56% yield) as a pale yellow solid. ^{1}H NMR (400 MHz, $CDCl_3$): δ 8.01–7.96 (m, 4H), 7.58–7.47 (m, 6H), 6.80 (s, 2H), 2.25 (s, 3H), 2.02 (s, 6H); ^{13}C NMR (100 MHz, $CDCl_3$): δ 220.23 (d, $J_{P\text{-}C}$ = 61.3 Hz), 140.68, 136.35 (d, $J_{P\text{-}C}$ = 39.7 Hz), 135.00, 132.47 (d, $J_{P\text{-}C}$ = 2.6 Hz), 131.99 (d, $J_{P\text{-}C}$ = 8.6 Hz), 129.85 (d, $J_{P\text{-}C}$ = 92.8 Hz), 129.00, 128.81 (d, $J_{P\text{-}C}$ = 11.6 Hz), 21.31, 19.78. ^{31}P NMR (162 MHz, $CDCl_3$): δ 13.78. MS (ESI) m/z: ($[M]^+$) Calcd for $C_{22}H_{21}O_2P$ 348, found 348. Melting Point: 82.4-86.3°C. This compound is known.[2]

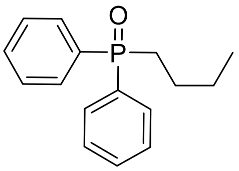

2-3a

Butyldiphenylphosphine oxide (**2-3a**). According to the general procedure A-3, purification by HPLC (standard conditions) afforded **2-3a** (255 mg, 99% yield) as a white solid; ^{1}H NMR (400 MHz, $CDCl_3$): δ 7.72–7.67 (m, 4H), 7.48–7.39 (m, 6H), 2.25–2.18 (m, 2H), 1.59–1.51 (m, 2H), 1.42–1.33 (m, 2H), 0.84 (t, 3H, J = 7.2 Hz); ^{13}C NMR (100 MHz, $CDCl_3$): δ 133.31 (d, $J_{P\text{-}C}$ = 97.2 Hz), 131.67 (d, $J_{P\text{-}C}$ = 2.4 Hz), 130.83 (d, $J_{P\text{-}C}$ = 9.2 Hz), 128.66 (d, $J_{P\text{-}C}$ = 11.4 Hz), 29.56 (d, $J_{P\text{-}C}$ = 71.8 Hz), 24.17 (d, $J_{P\text{-}C}$ = 15.0 Hz), 23.55 (d, $J_{P\text{-}C}$ = 3.8 Hz), 13.66; ^{31}P NMR (162 MHz, $CDCl_3$): δ 33.07. MS (ESI) m/z: ($[M]^+$) Calcd for $C_{16}H_{19}OP$ 258, Found 258; Melting Point: 91.5-93.2 °C. This compound is known and in agreement with the literature data.[3]

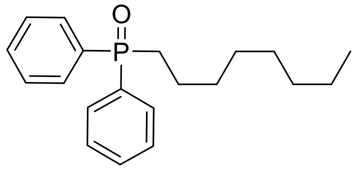

2-3b

Octyldiphenylphosphine oxide (2-3b). According to the general procedure A-3, purification by HPLC (standard conditions) afforded **2-3b** (310 mg, 99% yield) as a white solid; ^{1}H NMR (400 MHz, $CDCl_3$): δ 7.73–7.68 (m, 4H), 7.49-7.40 (m, 6H), 2.25–2.19 (m, 2H), 1.64–1.54 (m, 2H), 1.39–1.32 (m, 2H), 1.26–1.19 (m, 8H), 0.82 (t, 3H, J = 14.4 Hz); ^{13}C NMR (100 MHz, $CDCl_3$): δ 133.34 (d, $J_{P\text{-}C}$ = 157.0 Hz), 131.66 (d, $J_{P\text{-}C}$ = 2.1 Hz), 130.84 (d, $J_{P\text{-}C}$ = 9.4 Hz), 128.66 (d, $J_{P\text{-}C}$ = 11.6 Hz), 31.82, 31.05 (d, $J_{P\text{-}C}$ = 14.6 Hz), 30.18, 29.47, 29.28 (d, $J_{P\text{-}C}$ = 1.4 Hz), 22.66, 21.48 (d, $J_{P\text{-}C}$ = 3.8 Hz), 14.14; ^{31}P NMR (162 MHz, $CDCl_3$): δ 33.08 . MS (ESI) m/z: ([M]$^+$) Calcd for $C_{20}H_{27}OP$ 314, Found 314; Melting Point: 62.5-63.9°C. This compound is known and in agreement with the literature data.[4]

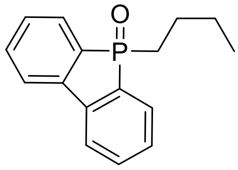

3'a

5-buylbenzo[b]phosphindole 5-oxide (3'a). According to the general procedure B, purification by HPLC (standard conditions) afforded **3'a** (170 mg, 74% yield) as a colorless oil; ^{1}H NMR (400 MHz, $CDCl_3$): δ 7.85–7.77 (m, 4H), 7.57 (t, 2H, J = 7.6 Hz), 7.44–7.40 (m, 2H), 2.11–2.04 (m, 2H), 1.57–1.47 (m, 2H), 1.40–1.30 (m, 2H), 0.84 (t, 3H, J = 14.4 Hz); ^{13}C NMR (100 MHz, $CDCl_3$): δ 141.27 (d, $J_{P\text{-}C}$ = 20.6 Hz), 133.19, 132.12 (d, $J_{P\text{-}C}$ = 100.2 Hz), 129.48 (d, $J_{P\text{-}C}$ = 9.2 Hz), 129.20 (d, $J_{P\text{-}C}$ = 10.6 Hz) , 121.27 (d, $J_{P\text{-}C}$ = 9.4 Hz), 30.10 (d, $J_{P\text{-}C}$ = 69.4 Hz), 24.17(d, $J_{P\text{-}C}$ = 3.0 Hz), 24.11 (d, $J_{P\text{-}C}$ = 22.4 Hz), 13.58; ^{31}P NMR (162 MHz, $CDCl_3$): δ 44.37. MS (ESI) m/z: ([M]$^+$) Calcd for $C_{16}H_{17}OP$ 256, Found 256. This compound is known and in agreement with the literature data.[5]

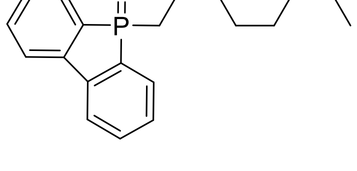

3'b

5-octylbenzo[b]phosphindole 5-oxide (3'b). According to the general procedure B, purification by HPLC (standard conditions) afforded **3'b** (224 mg, 80% yield) as a colorless oil;

^{1}H NMR (400 MHz, $CDCl_3$): δ 7.83–7.75 (m, 4H), 7.55 (t, 2H, J = 7.2 Hz), 7.42–7.37 (m, 2H), 2.09–2.01 (m, 2H), 1.56–1.46 (m, 2H), 1.30–1.17 (m, 10H), 0.82 (t, 3H, J = 6.9 Hz); ^{13}C NMR (100 MHz, $CDCl_3$): δ 141.25 (d, $J_{P\text{-}C}$ = 20.8 Hz), 133.23, 132.07 (d, $J_{P\text{-}C}$ = 100.4 Hz), 129.47 (d, $J_{P\text{-}C}$ = 9.4 Hz), 129.20 (d, $J_{P\text{-}C}$ = 10.6 Hz) , 121.30 (d, $J_{P\text{-}C}$ = 9.6 Hz), 31.773, 30.90 (d, $J_{P\text{-}C}$ = 15.2 Hz), 30.66, 29.97, 29.01 (d, $J_{P\text{-}C}$ = 1.6 Hz), 22.64, 22.13 (d, $J_{P\text{-}C}$ = 3.4 Hz), 14.13; ^{31}P NMR (162 MHz, $CDCl_3$): δ 44.36. . MS (ESI) m/z: ([M]$^+$) Calcd for $C_{20}H_{25}OP$ 312, Found 312; This compound is known and in agreement with the literature data.[6]

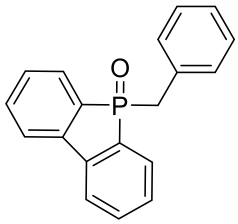

3'c

5-benzylbenzo[b]phosphindole 5-oxide (3'c). According to the general procedure B, purification by HPLC (standard conditions) afforded **3'c** (210 mg, 81% yield) as a white solid; ^{1}H NMR (400 MHz, $CDCl_3$): δ 7.69–7.66 (m, 2H), 7.57–7.49 (m, 4H), 7.36–7.31 (m, 2H), 7.18–7.17 (m, 3H), 7.05–7.03 (m, 2H), 3.41 (d, 2H, J = 15.2 Hz); ^{13}C NMR (100 MHz, $CDCl_3$): δ 141.11 (d, $J_{P\text{-}C}$ = 44.0 Hz), 133.34, 131.19 (d, $J_{P\text{-}C}$ = 102.2 Hz), 131.34 (d, $J_{P\text{-}C}$ = 7.8 Hz), 129.92 (d, $J_{P\text{-}C}$ = 14.2 Hz), 129.95, 129.00 (d, $J_{P\text{-}C}$ = 10.8 Hz) , 128.43 (d, $J_{P\text{-}C}$ = 2.4 Hz), 127.00 (d, $J_{P\text{-}C}$ = 3.2 Hz), 121.17 (d, $J_{P\text{-}C}$ = 9.8 Hz), 38.42 (d, $J_{P\text{-}C}$ = 64.2 Hz); ^{31}P NMR (162 MHz, $CDCl_3$): δ 40.31. MS (ESI) m/z: ([M]$^+$) Calcd. for $C_{19}H_{15}OP$ 290, Found 290. Melting Point: 129.7-136.1 °C. This compound is known and in agreement with the literature data.[7]

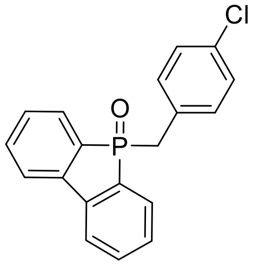

3'd

5-(4-chlorobenzyl)benzo[b]phosphindole 5-oxide (3'd). According to the general procedure B, purification by HPLC (standard conditions) afforded **3'd** (239 mg, 82% yield) as a white solid; ^{1}H NMR (400 MHz, $CDCl_3$): δ 7.70–7.68 (m, 2H), 7.59–7.51 (m, 4H), 7.37–7.33 (m, 2H), 7.14 (d, 2H, J = 8.4 Hz), 6.96–6.94 (m, 2H), 3.38 (d, 2H, J = 15.2 Hz); ^{13}C NMR (100 MHz, $CDCl_3$): δ

141.11 (d, J_{P-C} = 21.4 Hz), 133.49, 132.21 (d, J_{P-C} = 169.8 Hz), 131.29 (d, J_{P-C} = 14.9 Hz), 130.15 (d, J_{P-C} = 38.5 Hz), 129.88, 129.79, 129.10 (d, J_{P-C} = 10.5 Hz) , 128.55 (d, J_{P-C} = 2.1 Hz), 121.27 (d, J_{P-C} = 9.8 Hz), 37.81 (d, J_{P-C} = 64.1 Hz); ^{31}P NMR (162 MHz, $CDCl_3$): δ 40.02. MS (ESI) m/z: ($[M]^+$) Calcd. for $C_{16}H_{14}ClOP$ 324, Found 324. Melting Point: 75.6-80.3 °C. This is a new compound.

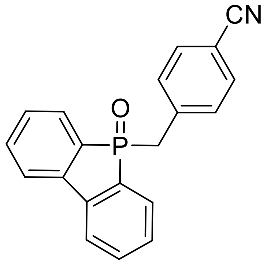

3'e

4-((5-oxidobenzo[b]phosphindol-5-yl)methyl)benzonitrile (3'e). According to the general procedure B, purification by HPLC (standard conditions) afforded **3'e** (146 mg, 52% yield) as a white solid; ^{1}H NMR (400 MHz, $CDCl_3$): δ 7.70–7.67 (m, 2H), 7.59–7.53 (m, 4H), 7.43 (d, 2H, J = 8.0 Hz), 7.39–7.35 (m, 2H), 7.11–7.09 (m, 2H), 3.48 (d, 2H, J = 15.6 Hz); ^{13}C NMR (100 MHz, $CDCl_3$): δ 141.04 (d, J_{P-C} = 21.8 Hz), 137.27 (d, J_{P-C} = 7.9 Hz), 133.77, 132.05 (d, J_{P-C} = 2.5 Hz), 130.06 (d, J_{P-C} = 5.1 Hz), 129.75 (d, J_{P-C} = 9.0 Hz), 129.25 (d, J_{P-C} = 10.7 Hz) , 121.38 (d, J_{P-C} = 10.1 Hz), 118.68, 110.95 (d, J_{P-C} = 3.5 Hz), 38.91 (d, J_{P-C} = 62.2 Hz); ^{31}P NMR (162 MHz, $CDCl_3$): δ 39.22. MS (ESI) m/z: ($[M]^+$) Calcd. for $C_{20}H_{14}NOP$ 315, Found 315. Melting Point: 167.3-183.5 °C. This is a new compound.

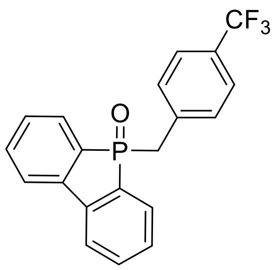

3'f

5-(4-(trifluoromethyl)benzyl)benzo[b]phosphindole 5-oxide (3'f). According to the general procedure B, purification by HPLC (standard conditions) afforded **3'f** (177 mg, 55% yield) as a white solid; ^{1}H NMR (400 MHz, $CDCl_3$): δ 7.69–7.66 (m, 2H), 7.57–7.50 (m, 4H), 7.43 (d, 2H, J = 8.4 Hz), 7.36–7.33 (m, 2H), 7.15 (d, 2H, J = 6.8 Hz), 3.44 (d, 2H, J = 15.2 Hz); ^{13}C NMR (100 MHz, $CDCl_3$): δ 141.04 (d, J_{P-C} = 21.6 Hz), 135.74 (d, J_{P-C} = 7.9 Hz), 133.62 (d, J_{P-C} = 1.5 Hz),

131.22, 130.23 (d, J_{P-C} = 5.2 Hz), 129.76 (d, J_{P-C} = 9.0 Hz), 129.16 (d, J_{P-C} = 10.7 Hz) , 125.28 (d, J_{P-C} = 6.4 Hz), 125.28, 121.33 (d, J_{P-C} = 10.1 Hz), 38.48 (d, J_{P-C} = 62.9 Hz); ^{31}P NMR (162 MHz, $CDCl_3$): δ 39.23. MS (ESI) m/z: ($[M]^+$) Calcd. for $C_{20}H_{14}F_3OP$ 358, Found 358. Melting Point: 155.1-157.0 °C. This is a new compound.

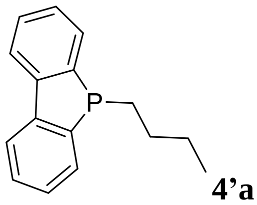

4'a

5-butyl-5H-benzo[b]phosphindole (4'a). According to the general procedure C, purification by HPLC (standard conditions) afforded **4'a** (158 mg, 73% yield) as a yellow oil; ^{1}H NMR (400 MHz, $CDCl_3$): δ 7.96–7.91 (m, 2H), 7.78–7.71 (m, 2H), 7.50–7.44 (m, 2H), 7.39–7.34 (m, 2H), 1.82–1.78 (m, 2H), 1.44–1.31 (m, 4H), 0.87 (t, 2H, J = 13.6 Hz); ^{13}C NMR (100 MHz, $CDCl_3$): δ 143.84, 143.39 (d, J_{P-C} = 5.3 Hz), 130.00 (d, J_{P-C} = 20.1 Hz), 128.35, 127.17 (d, J_{P-C} = 7.4 Hz), 121.44, 29.49 (d, J_{P-C} = 17.7 Hz) , 28.12 (d, J_{P-C} = 8.0 Hz), 24.23 (d, J_{P-C} = 10.5 Hz), 13.79. ^{31}P NMR (162 MHz, $CDCl_3$): δ -13.66. MS (ESI) m/z: ($[M]^+$) Calcd for $C_{16}H_{17}P$ 240, Found 240. This compound is known and in agreement with the literature data.[8]

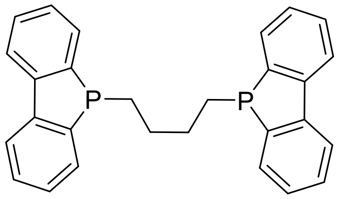

4'b

1,4-bis(5H-benzo[b]phosphindol-5-yl)butane (4'b). According to the general procedure C (note: 0.4 mmol 1,4-dibromobutane was added), purification by HPLC (standard conditions) afforded **4'b** (152 mg, 80% yield) as a yellow oil; ^{1}H NMR (400 MHz, $CDCl_3$): δ 7.92 (d, 4H, J = 8.0 Hz), 7.69–7.66 (m, 4H), 7.47–7.43 (m, 4H), 7.35–7.31 (m, 4H), 1.70 (t, 4H, J = 8.0 Hz), 1.39–1.33 (m, 4H) ; ^{13}C NMR (100 MHz, $CDCl_3$): δ 143.85, 143.0 (d, J_{P-C} = 5.5 Hz), 129.95 (d, J_{P-C} = 21.4 Hz), 128.40, 127.19 (d, J_{P-C} = 7.6 Hz), 121.41, 29.20 (d, J_{P-C} = 18.6 Hz), 27.26 (t, J_{P-C} = 8.9 Hz). ^{31}P NMR (162 MHz, $CDCl_3$): δ -13.82. MS (ESI) m/z: ($[M]^+$) Calcd. for $C_{27}H_{22}P_2$ 408, Found 408. This is a new compound.

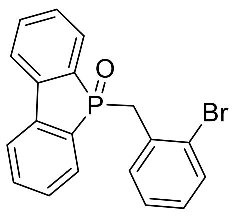

4’c

5-(2-bromobenzyl)benzo[b]phosphindole 5-oxide (**4’c**). According to the general procedure C, the product was oxidized by 1 mL 30 % H_2O_2 before isolation, purification by HPLC (standard conditions) afforded **4’c** (274 mg, 83% yield) as a yellow oil. ^1H NMR (400 MHz, $CDCl_3$): δ 7.71–7.68 (m, 2H), 7.61–7.52 (m, 4H), 7.40 (d, 1H, J = 8.0 Hz), 7.36–7.32 (m, 3H), 7.17 (t, 1H, J = 7.6 Hz), 7.06–7.02 (m, 1H), 3.69 (d, 2H, J = 15.6 Hz); ^{13}C NMR (100 MHz, $CDCl_3$): δ 141.10 (d, $J_{P\text{-}C}$ = 21.9 Hz), 133.44 (d, $J_{P\text{-}C}$ = 1.5 Hz), 132.91 (d, $J_{P\text{-}C}$ = 2.4 Hz), 131.85 (d, $J_{P\text{-}C}$ = 31.4 Hz), 131.60 (d, $J_{P\text{-}C}$ = 4.5 Hz), 130.61, 129.96 (d, $J_{P\text{-}C}$ = 8.9 Hz), 129.07 (d, $J_{P\text{-}C}$ = 10.7 Hz), 128.65 (d, $J_{P\text{-}C}$ = 3.4 Hz), 127.47 (d, $J_{P\text{-}C}$ = 2.8 Hz), 125.14 (d, $J_{P\text{-}C}$ = 6.7 Hz), 121.14 (d, $J_{P\text{-}C}$ = 10.1 Hz), 37.97 (d, $J_{P\text{-}C}$ = 63.8 Hz); ^{31}P NMR (162 MHz, $CDCl_3$): δ 39.63. MS (ESI) m/z: ($[M]^+$) Calcd. for $C_{19}H_{14Br}OP$ 368, Found 368. This is a new compound.

Supplementary References

(1) Lhermet, R., Moser, E., Jeanneau, E., Olivier-Bourbigou, H., & Breuil, P. A. R. Outer-Sphere reactivity shift of secondary phosphine oxide‐based nickel complexes: from ethylene hydrophosphinylation to oligomerization. *Chem. Eur J.* **23**, 7433-7437 (2017).

(2) (a) Lechtken, P., Buethe, I., Jacobi, M., & Trimborn, W. *US Patent* 4,298,738 (1981); (b) Wang Z., Wang Y., Wang Y., Song H. Preparation method of acyl phosphine oxide compound. *CN Patent* 103159796 (2011).

(3) Huang, T., Chen, T., & Han, L. B. Oxidative dephosphorylation of benzylic phosphonates with dioxygen generating symmetrical trans-stilbenes. *J. Org. Chem.* **83**, 2959-2965 (2018).

(4) Wang, F., Qu, M., Chen, F., Xu, Q., & Shi, M. Ph_2PI as a reduction/phosphination reagent: providing easy access to phosphine oxides. *Chem. Comm.* **48**, 8580-8582 (2012).

(5) Vedejs, E., & Marth, C. DBP ylides: Wittig reagents for synthesis of E-alkenes from aldehydes. *Tetrahedron Lett.* **28**, 3445-3448 (1987).

(6) https://pubchem.ncbi.nlm.nih.gov/compound/68843154. PubChem CID: 68843154

(7) (a) Allen, D. W., & Hutley, B. G. The chemistry of heteroarylphosphorus compounds, Part XIII [1] some reactions of 5-(2-thienyl) dibenzophosphole and its quaternary salts. *Zeitschrift für Naturforschung B*, **34**, 1116-1120 (1979). (b) Ezzell, B. R., & Freedman, L. D. Synthesis and cleavage reactions of some dibenzophosphole derivatives. *J. Org. Chem.* **34**, 1777-1780 (1969).

(8) Cornforth, J., Cornforth, R. H., & Gray, R. T. Synthesis of substituted dibenzophospholes. Part 1. *J. Chem. Soc., Perkin Trans. 1,* 2289-2297 (1982).

Supplementary Note 1

The chemical waste triphenylphosphine oxide $Ph_3P(O)$ was predominantly generated from the chemical industries as an end-up compound of triphenylphoshine Ph_3P. Ph_3P is the most popularly used organic phosphorus compound, and more than thousands of tons of this compound is manufactured every year (https://hpvchemicals.oecd.org/UI/handler.axd?id=898195ab-9e1a-44f3-9f08-d823481a2c40). It is used in several famous organic synthetic reactions such as the Mitsunobu, Staudinger, Wittig, Rauhut-Currier and Appeal reactions to produce vitamins, pharmaceuticals and agrochemicals. Triphenylphosphine is also the most important ligand for organometal catalysts, as exemplified by the Wilkinson catalyst $RhCl(PPh_3)_3$ for hydrogenation, the palladium catalyst $Pd(PPh_3)_4$ for couplings, and the metal catalysts for hydroformylation producing aldehydes (the Oxo synthesis) etc. These Ph_3P eventually ends up as $Ph_3P(O)$.

Copies of ^{1}H NMR, ^{31}P NMR and ^{13}C NMR spectra

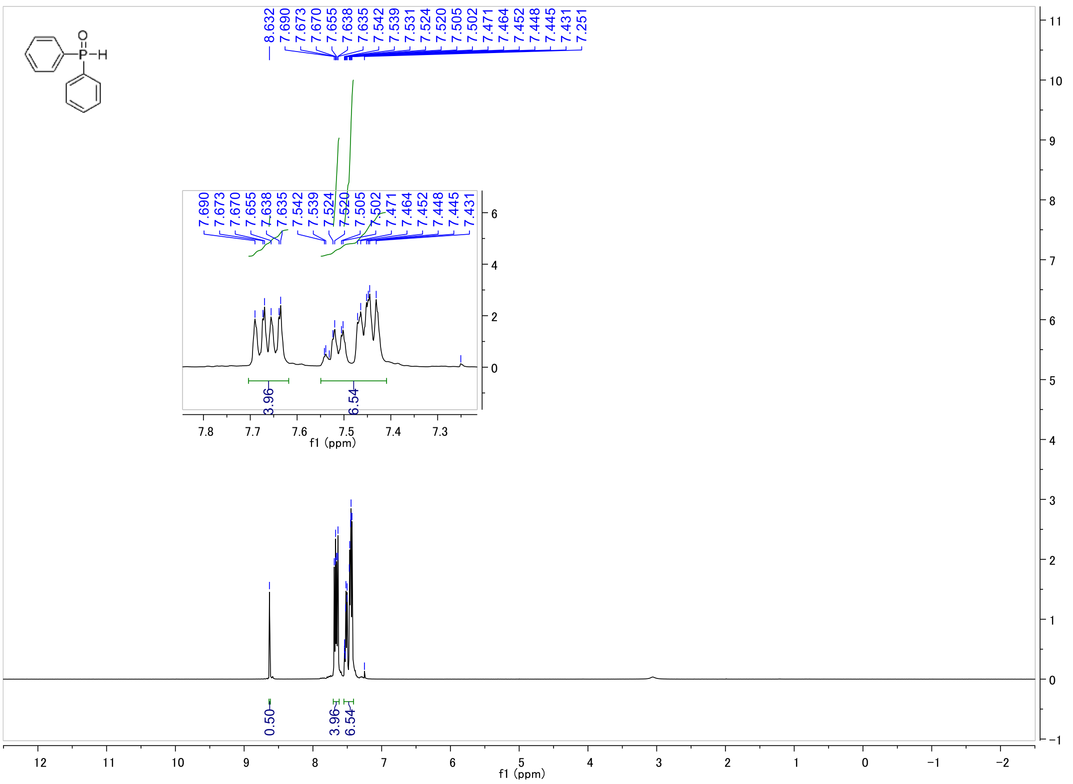

Supplementary Figure 12. ^{1}H NMR spectra of **2-1**

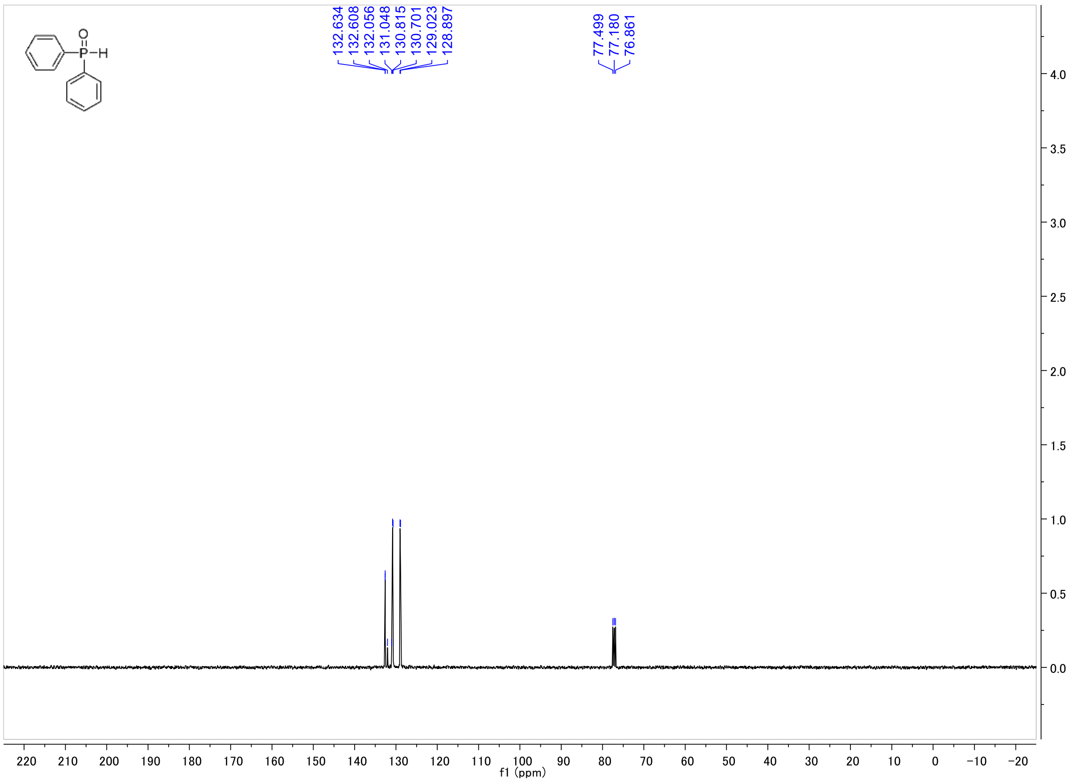

Supplementary Figure 13. ^{13}C NMR spectra of **2-1**

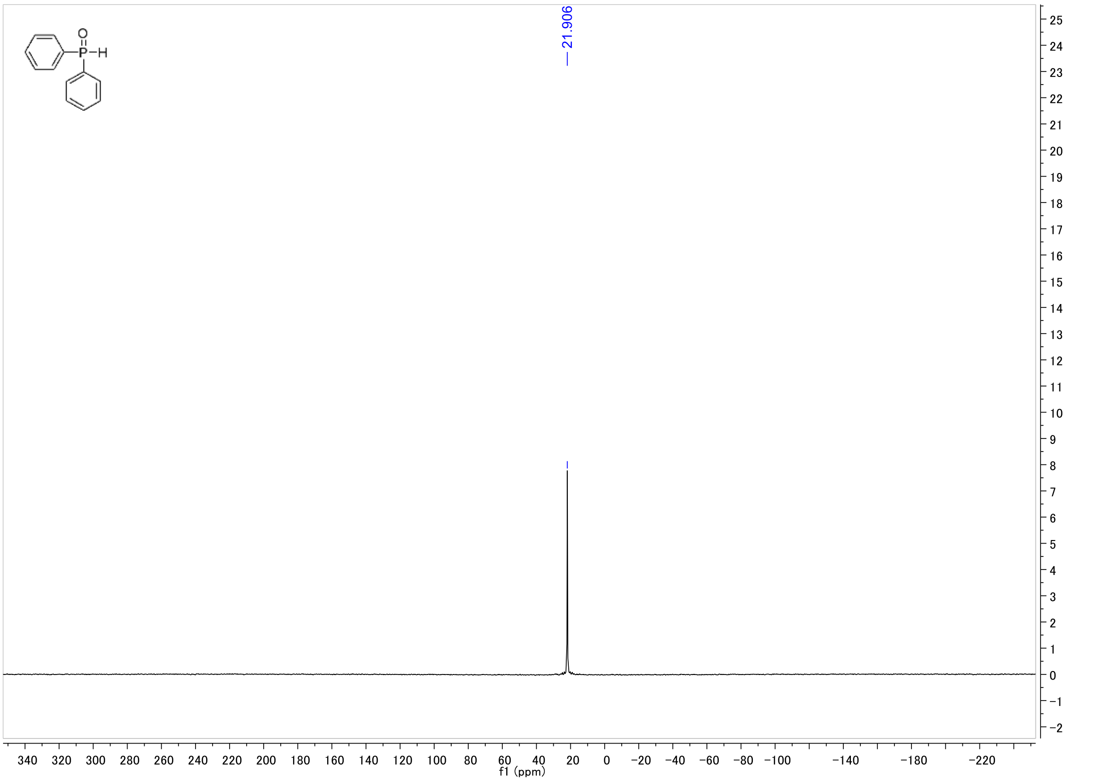

Supplementary Figure 14. ^{31}P NMR spectra of **2-1**

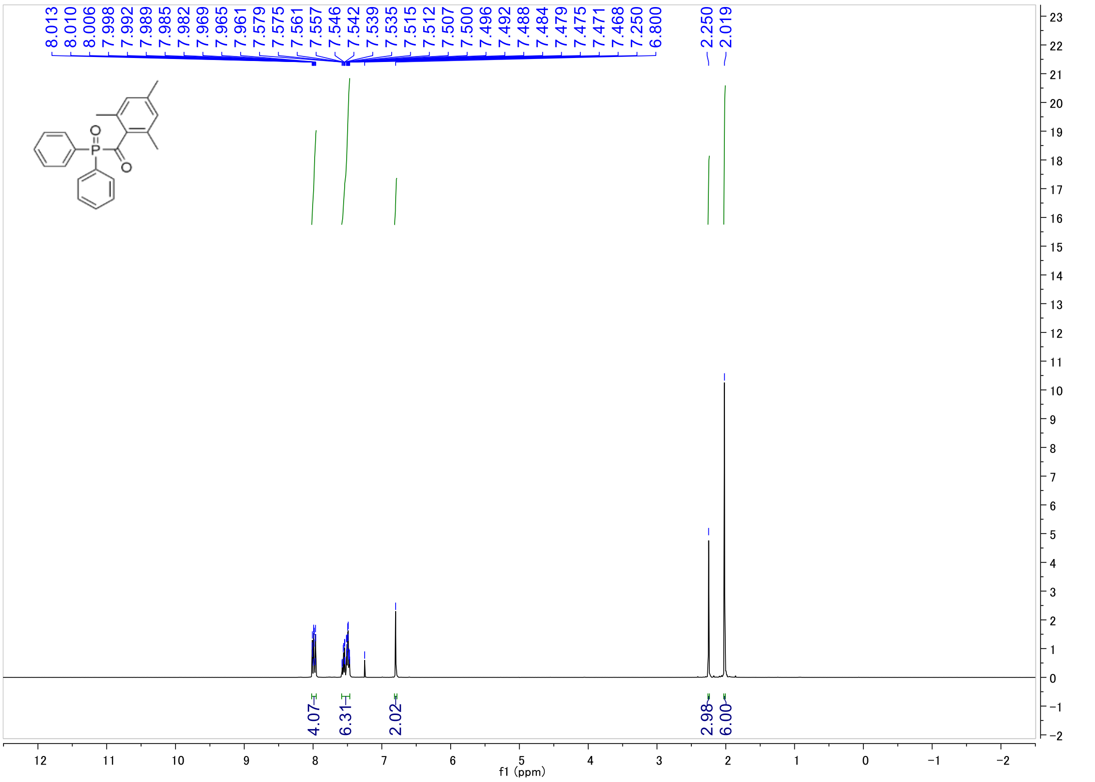

Supplementary Figure 15. ^{1}H NMR spectra of **2-2**

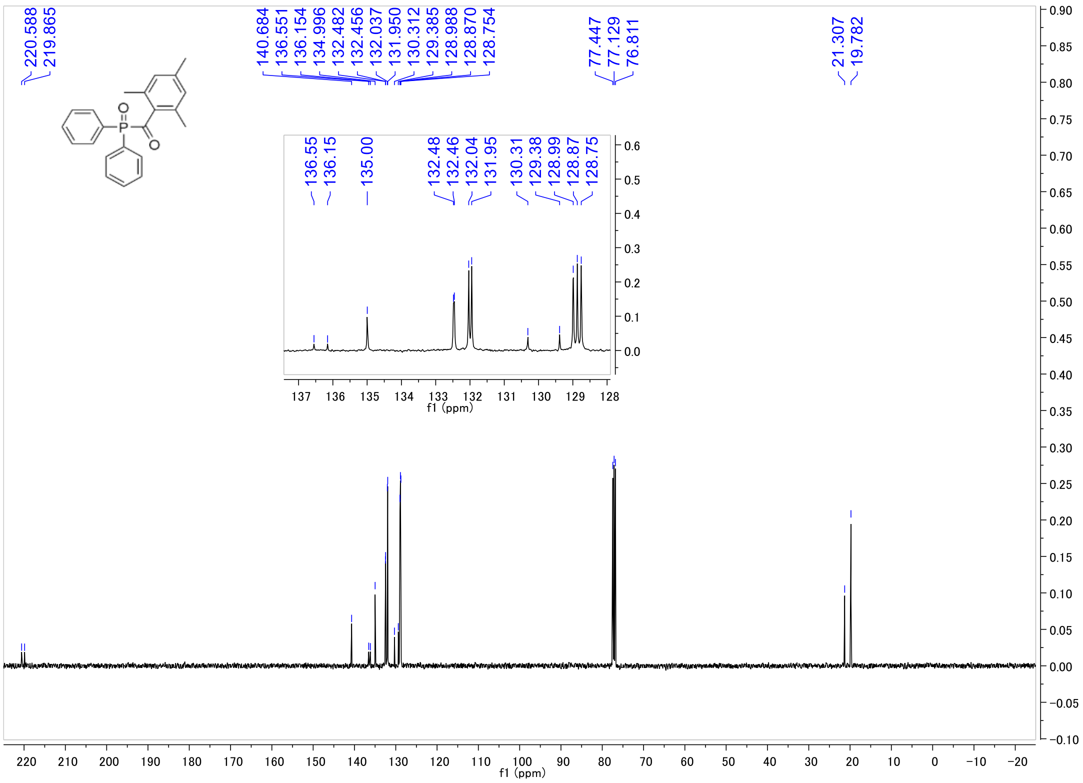

Supplementary Figure 16. ^{13}C NMR spectra of **2-2**

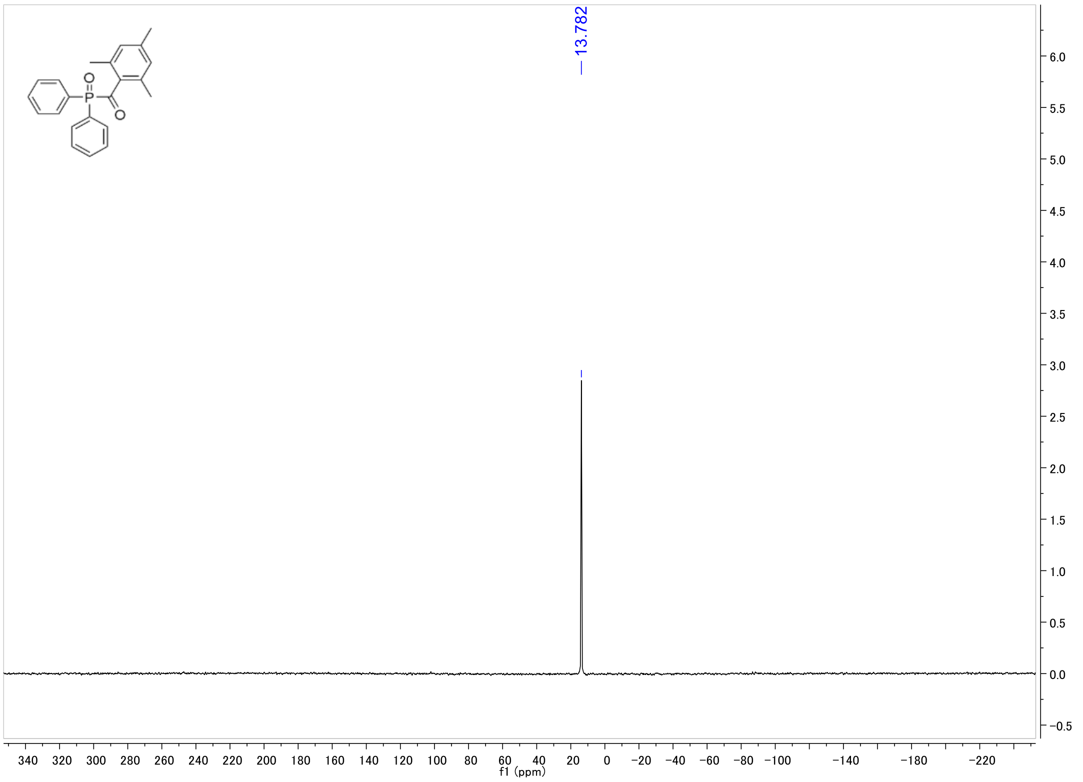

Supplementary Figure 17. ^{31}P NMR spectra of **2-2**

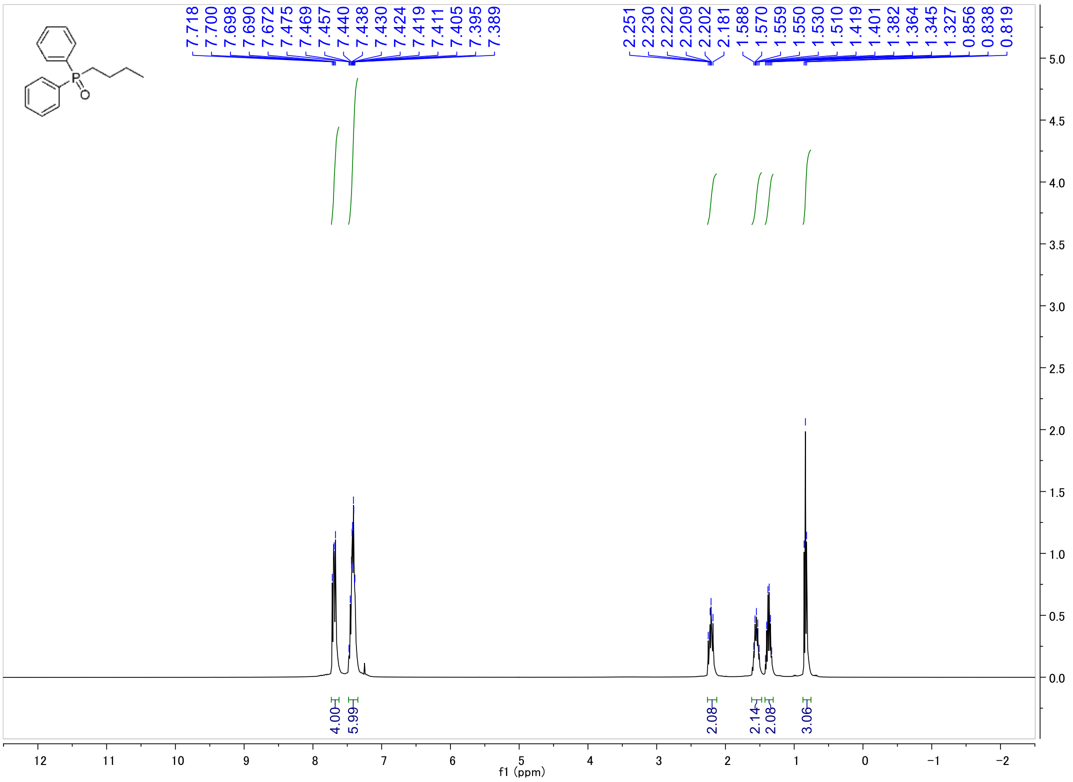

Supplementary Figure 18. ^{1}H NMR spectra of **2-3a**

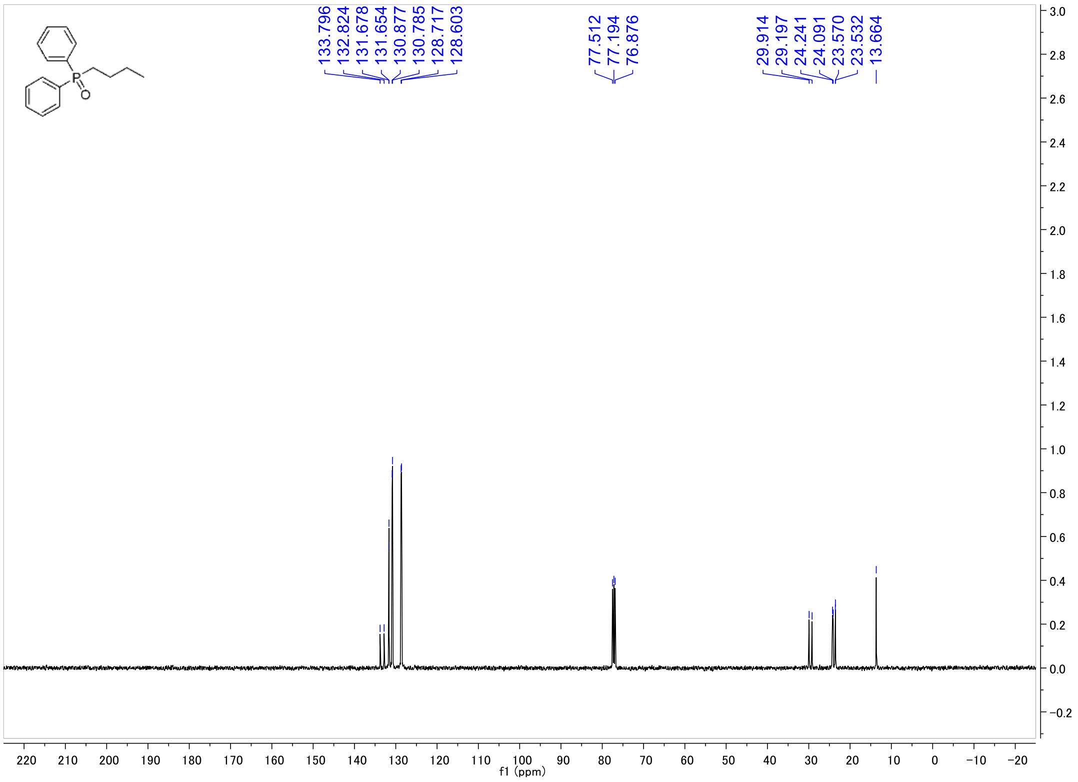

Supplementary Figure 19. ^{13}C NMR spectra of **2-3a**

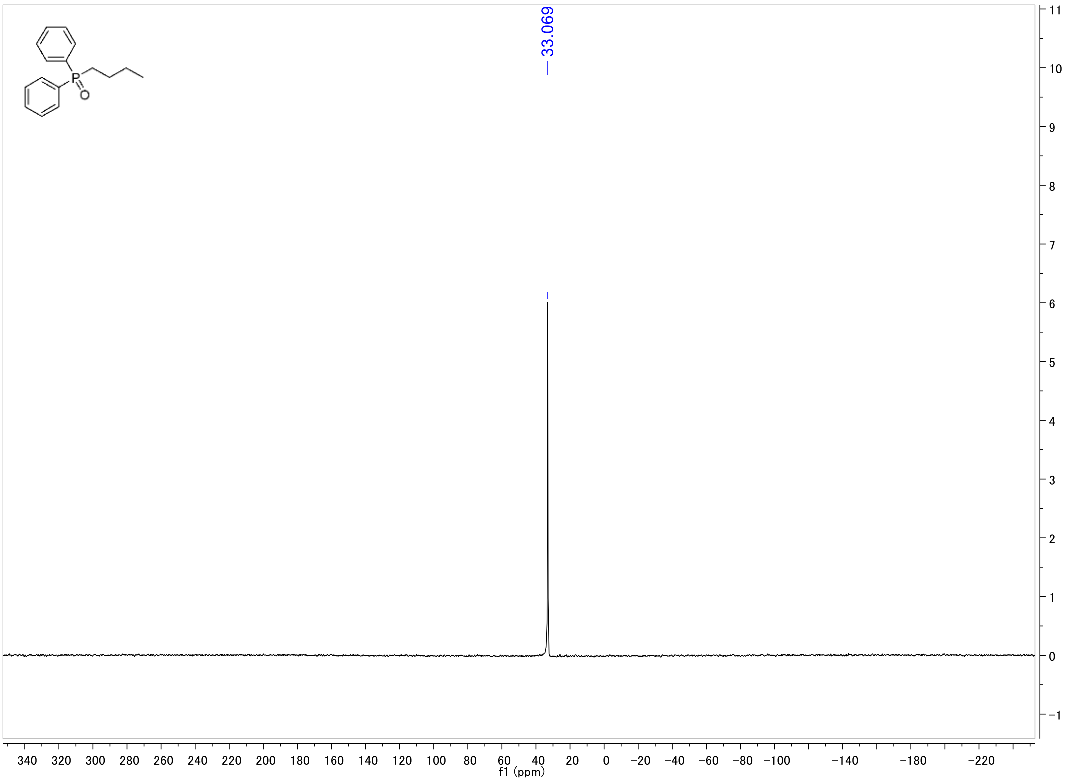

Supplementary Figure 20. ^{31}P NMR spectra of **2-3a**

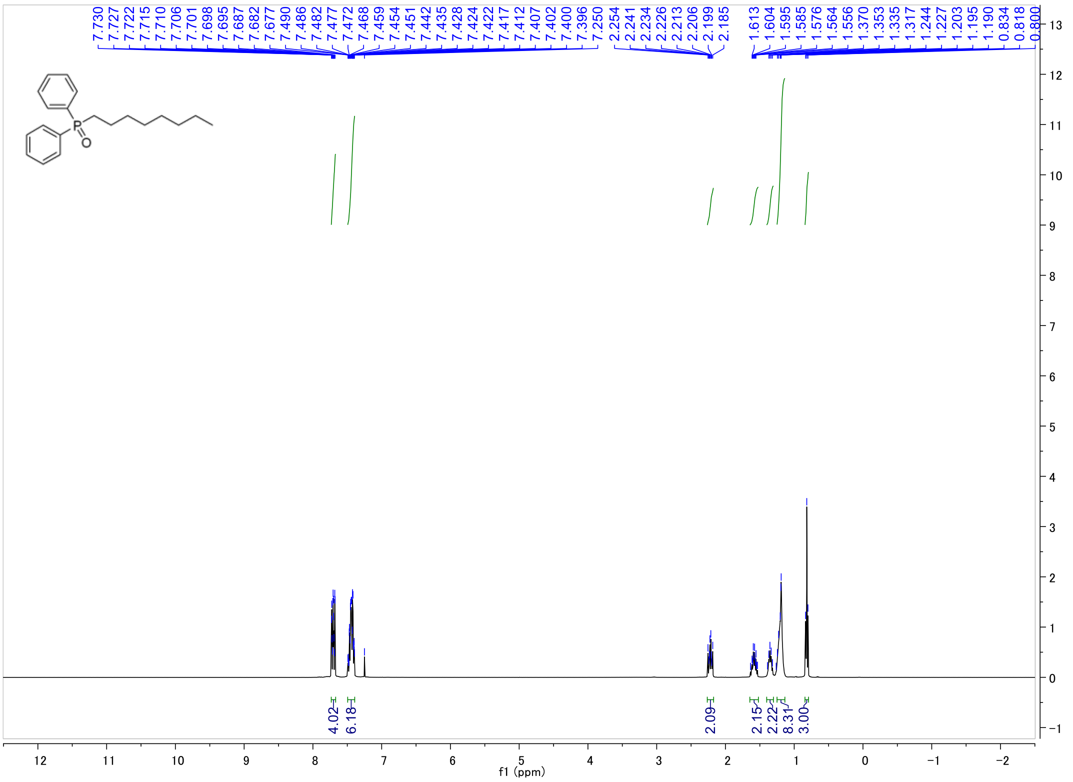

Supplementary Figure 21. ^{1}H NMR spectra of **2-3b**

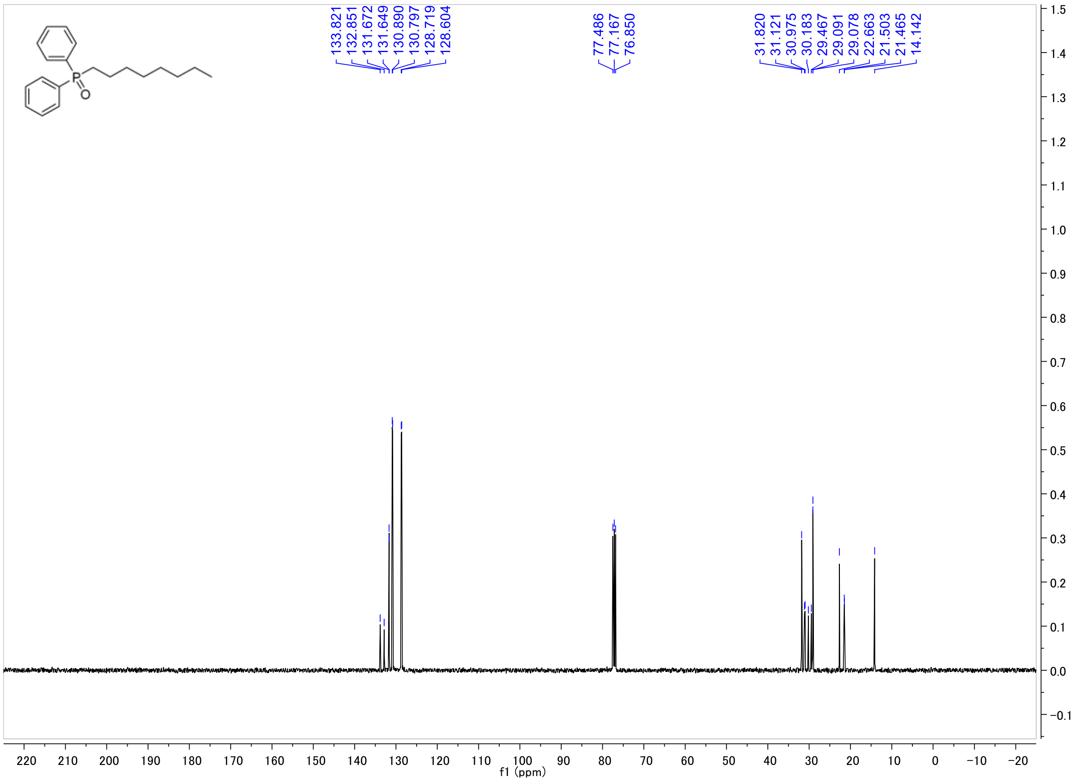

Supplementary Figure 22. ^{13}C NMR spectra of **2-3b**

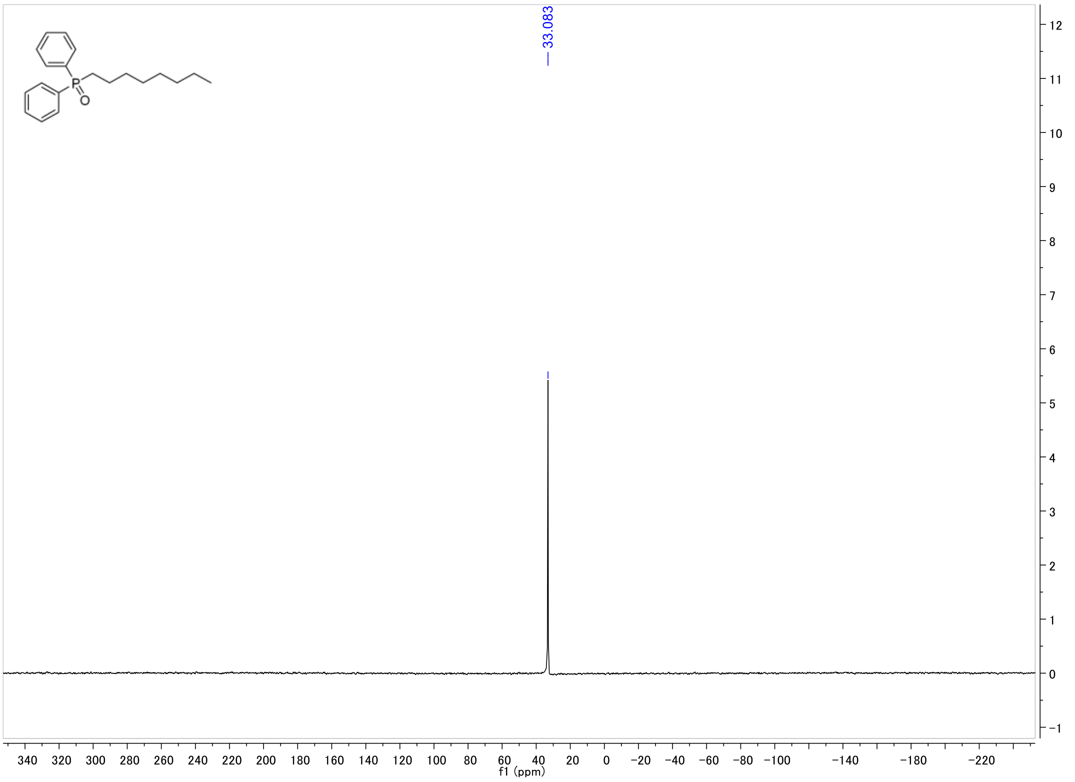

Supplementary Figure 23. ^{31}P NMR spectra of **2-3b**

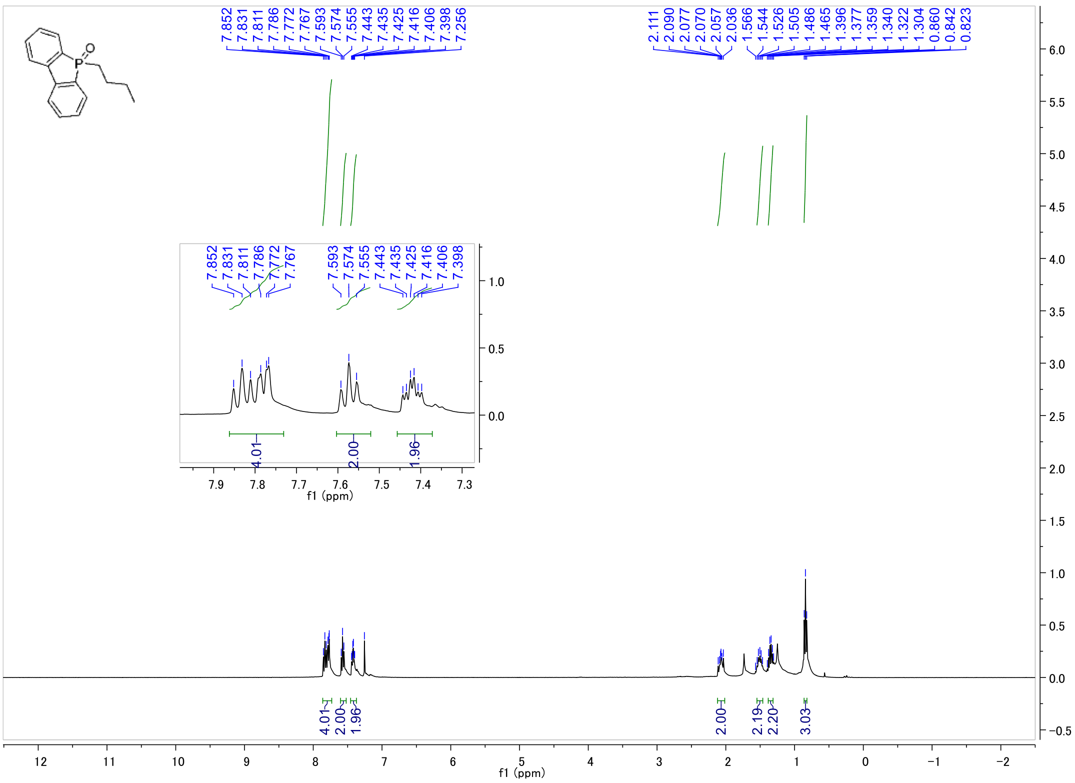

Supplementary Figure 24. ^{1}H NMR spectra of **3’a**

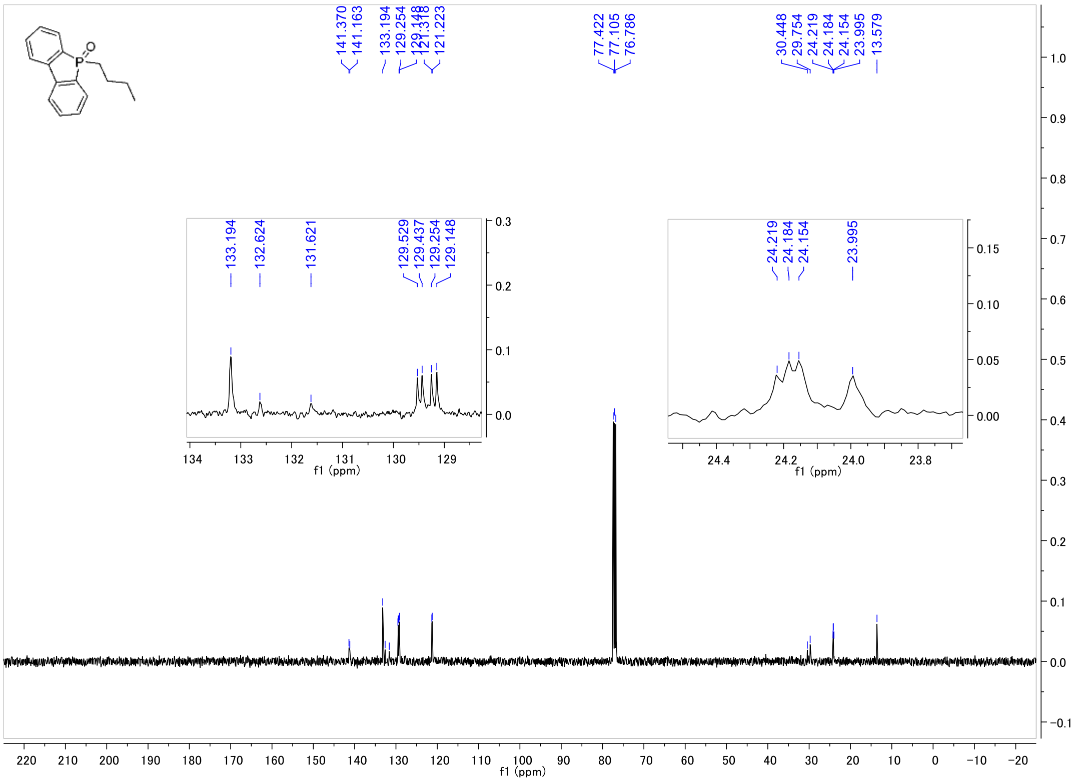

Supplementary Figure 25. ^{13}C NMR spectra of **3’a**

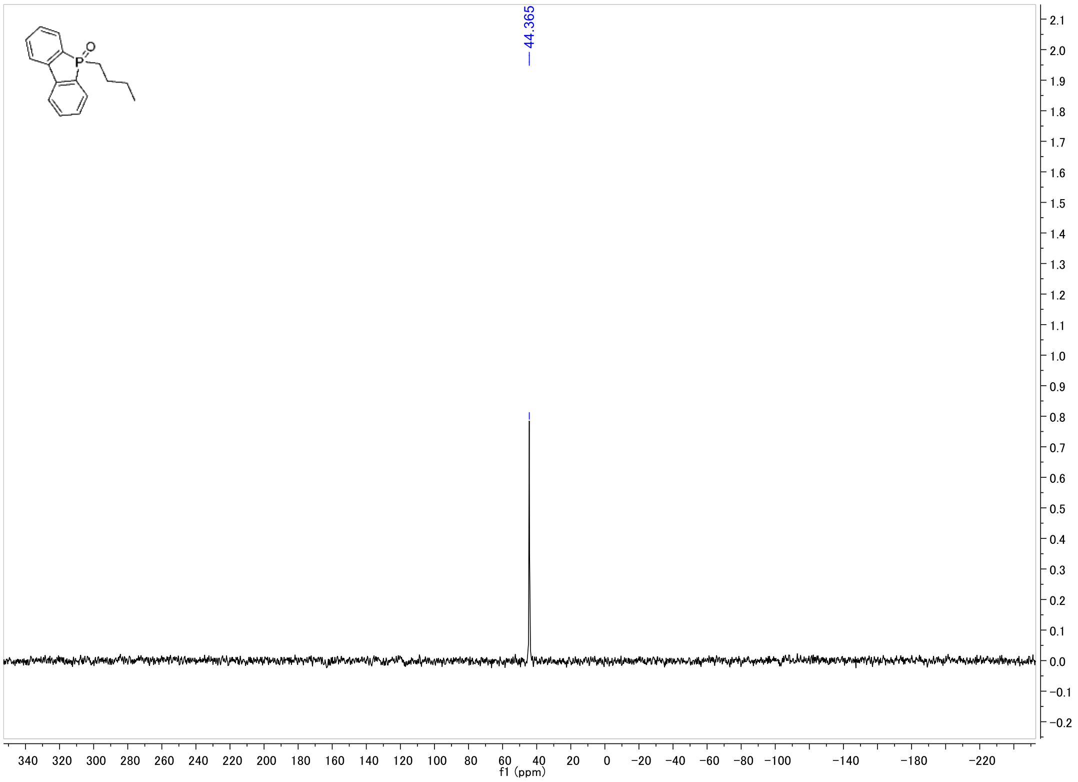

Supplementary Figure 26. ^{31}P NMR spectra of **3'a**

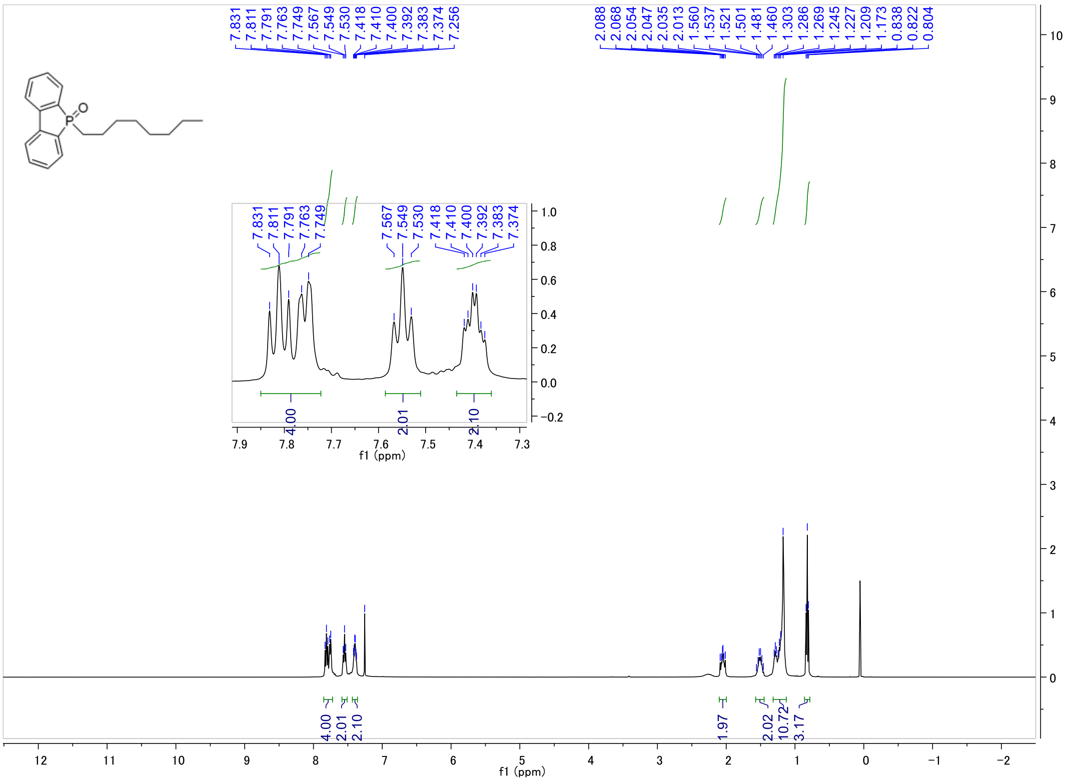

Supplementary Figure 27. ^{1}H NMR spectra of **3'b**

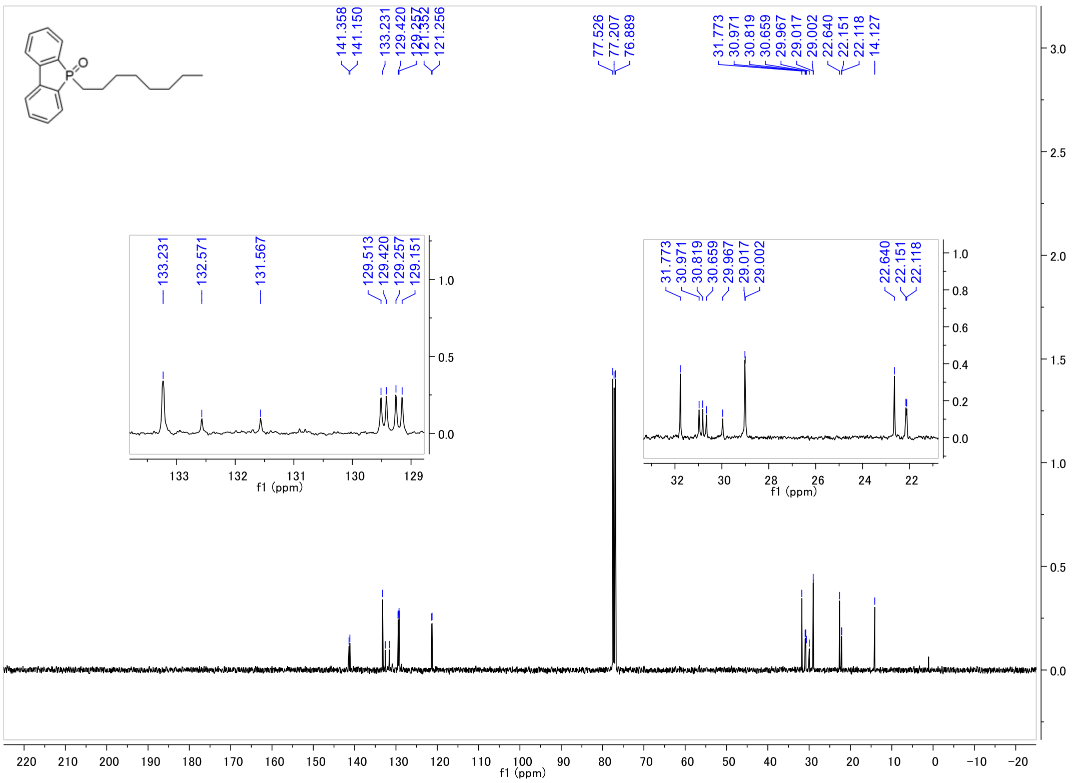

Supplementary Figure 28. ^{13}C NMR spectra of **3'b**

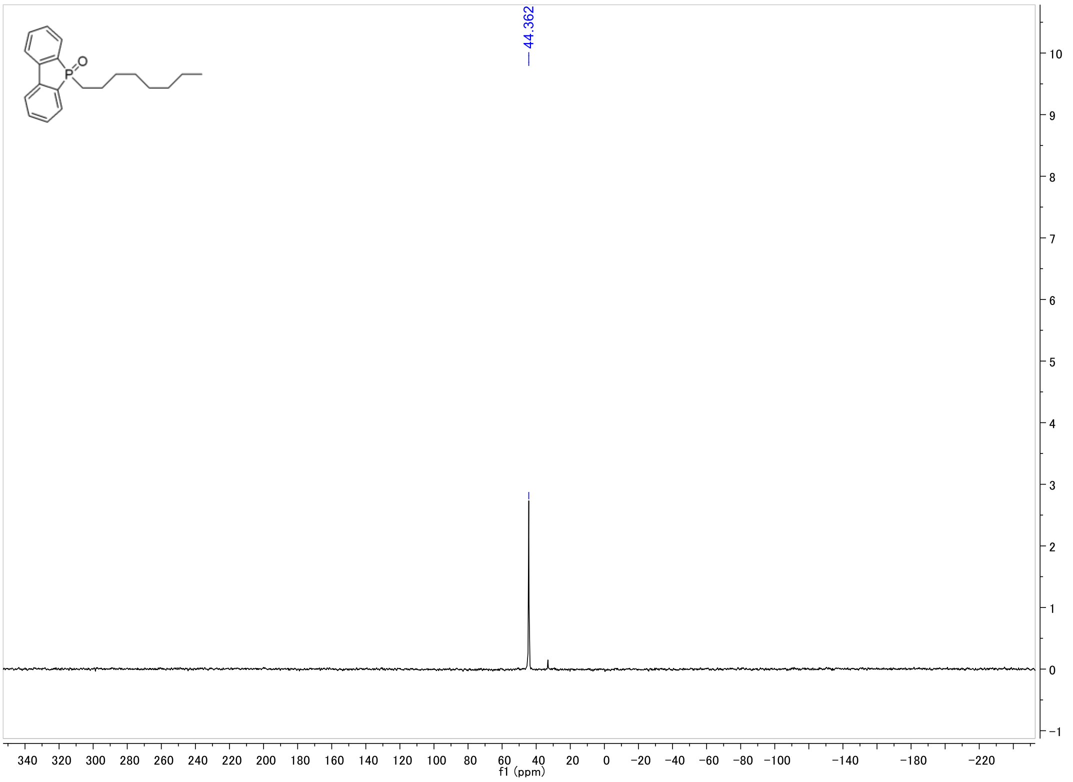

Supplementary Figure 29. ^{31}P NMR spectra of **3'b**

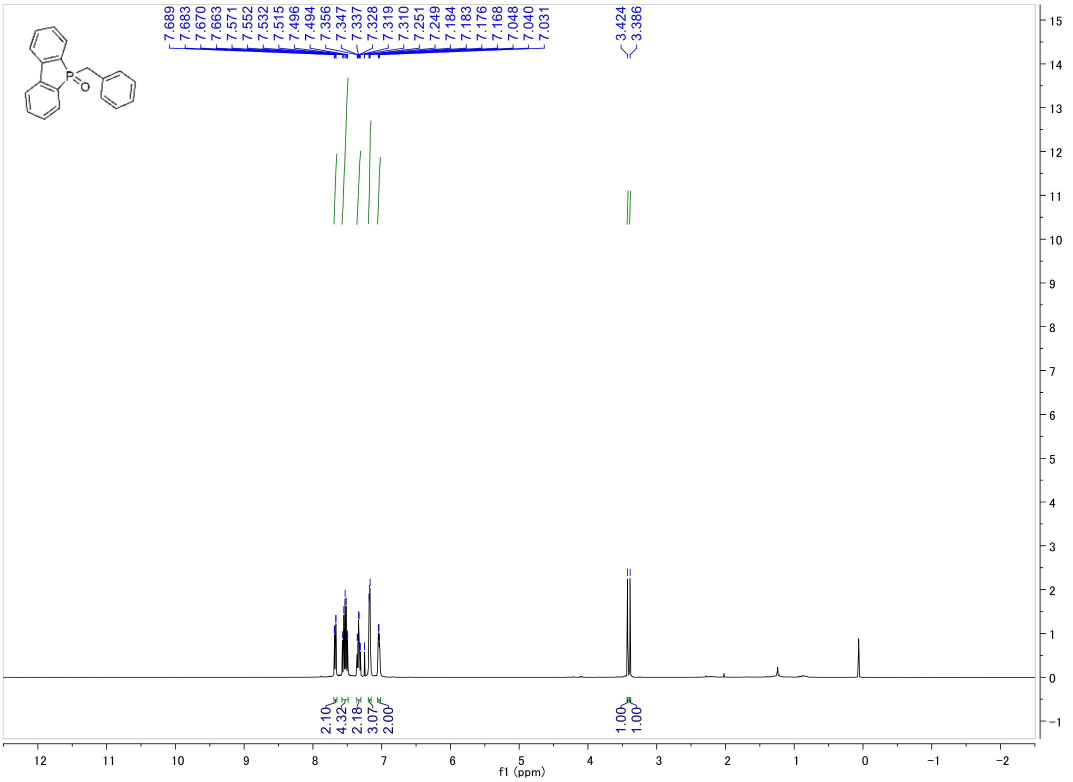

Supplementary Figure 30. ^{1}H NMR spectra of **3’c**

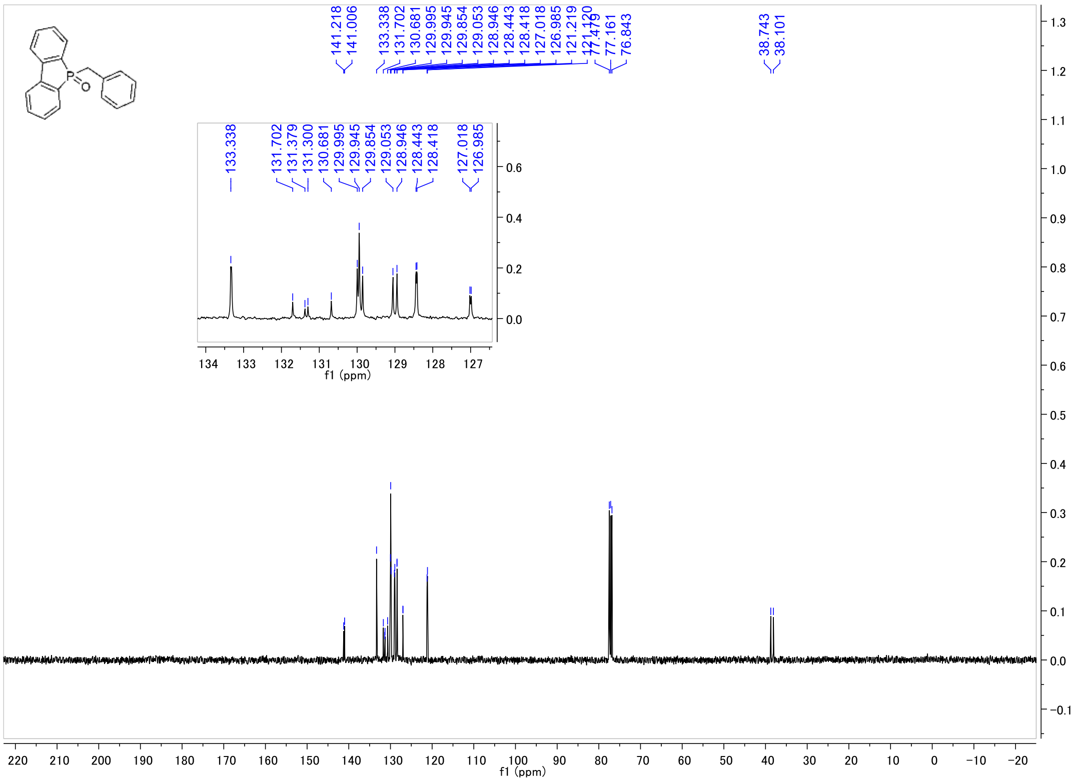

Supplementary Figure 31. ^{13}C NMR spectra of **3’c**

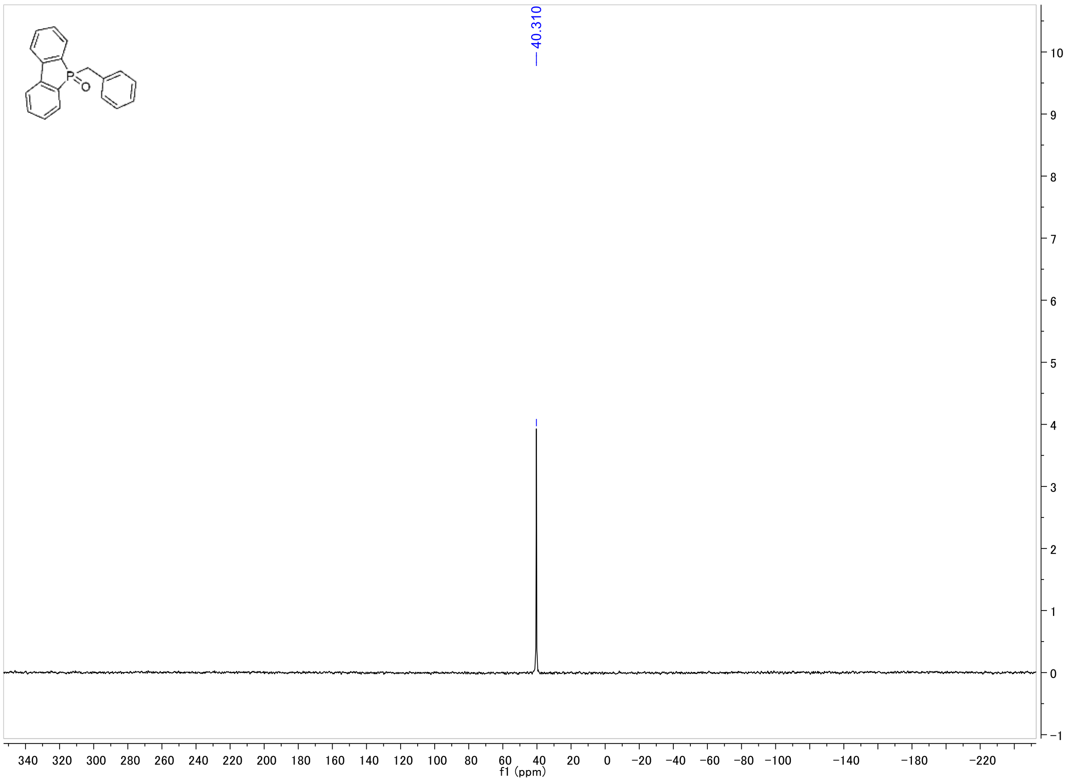

Supplementary Figure 32. ^{31}P NMR spectra of **3’c**

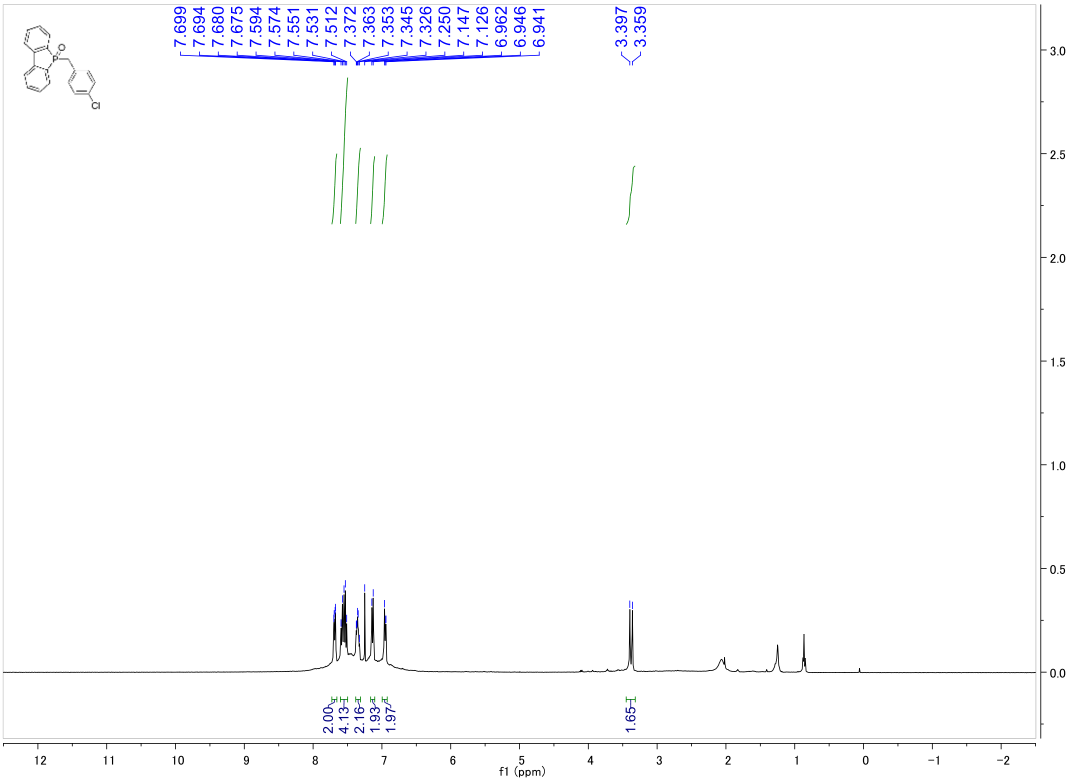

Supplementary Figure 33. ^{1}H NMR spectra of **3’d**

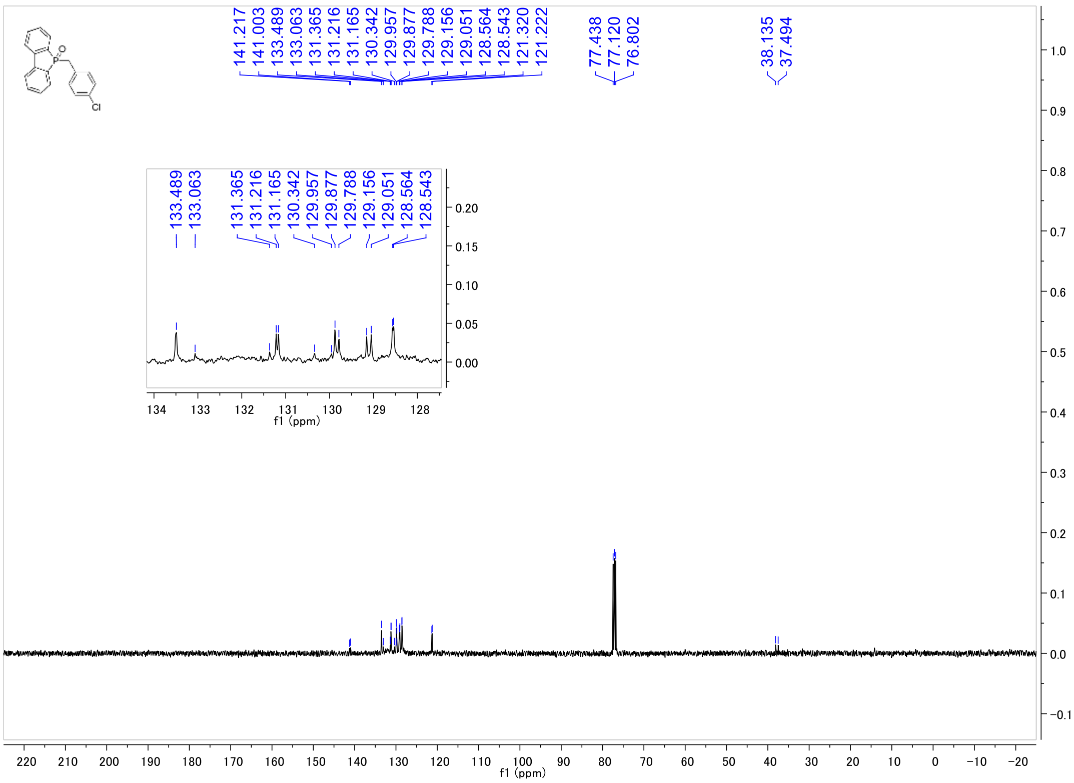

Supplementary Figure 34. ^{13}C NMR spectra of **3’d**

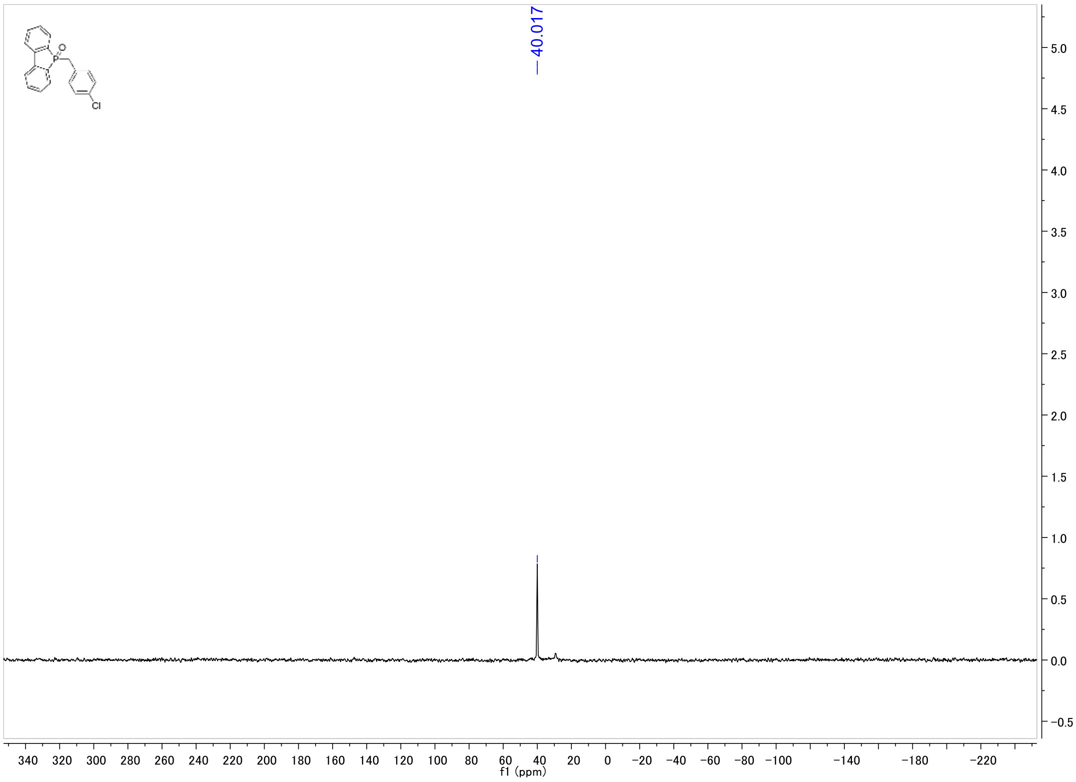

Supplementary Figure 35. ^{31}P NMR spectra of **3’d**

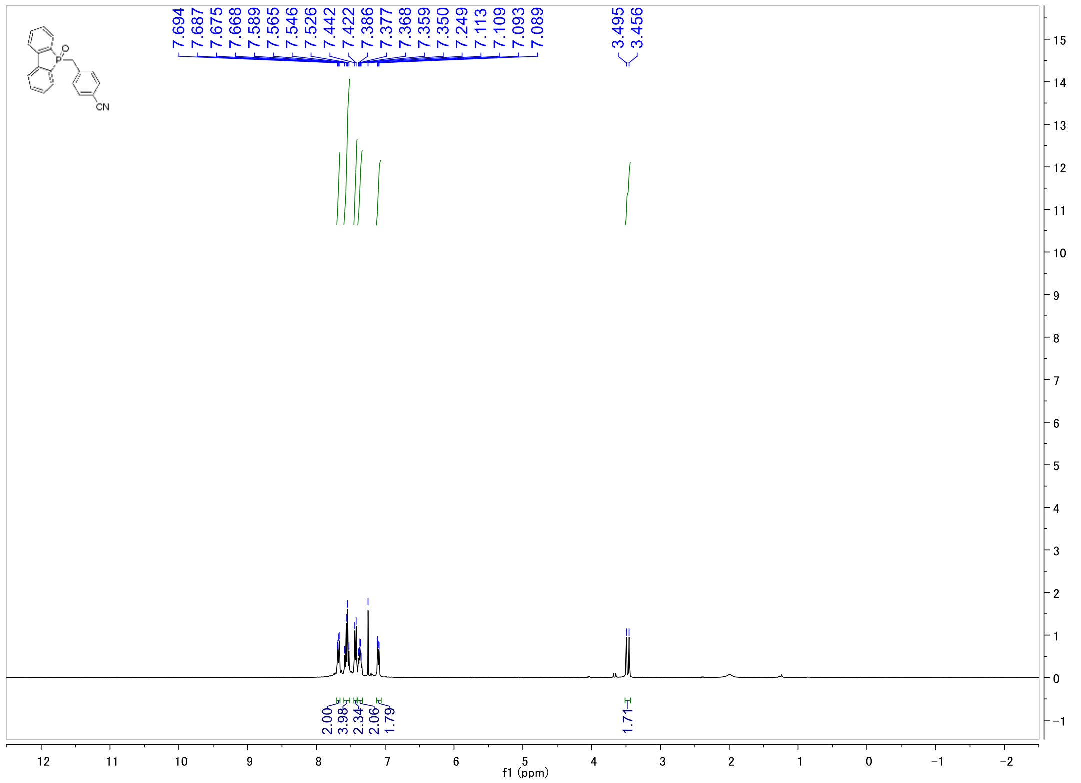

Supplementary Figure 36. ^{1}H NMR spectra of **3’e**

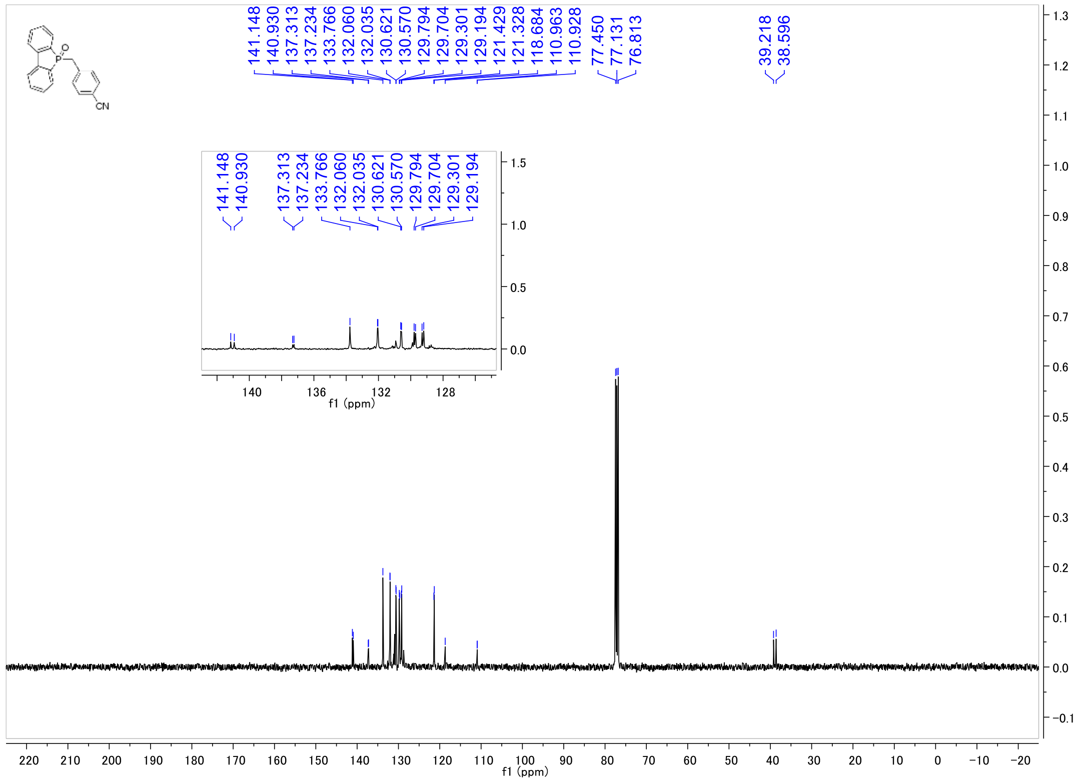

Supplementary Figure 37. ^{13}C NMR spectra of **3’e**

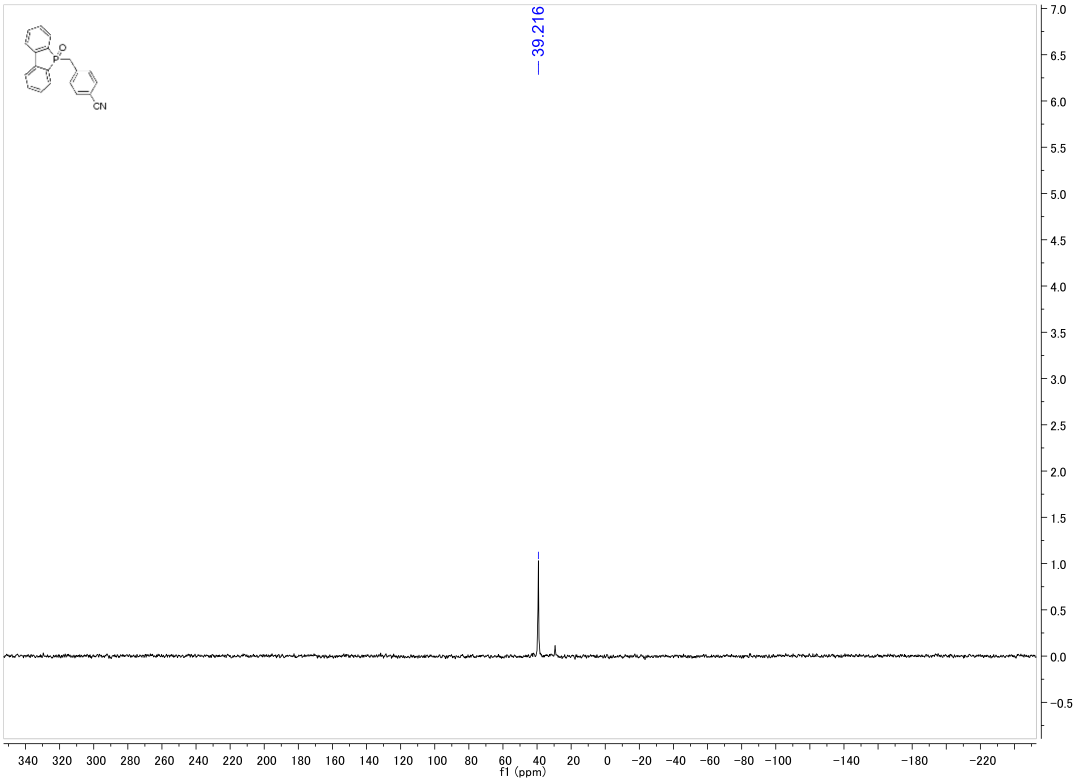

Supplementary Figure 38. ^{31}P NMR spectra of **3’e**

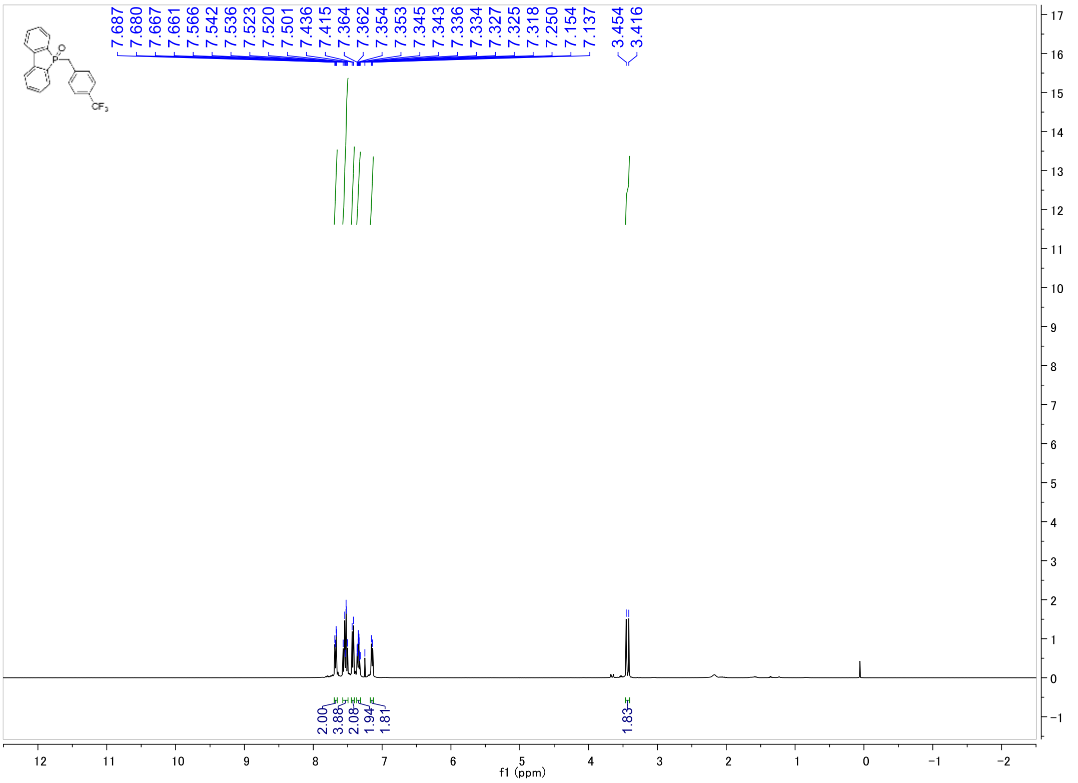

Supplementary Figure 39. ^{1}H NMR spectra of **3’f**

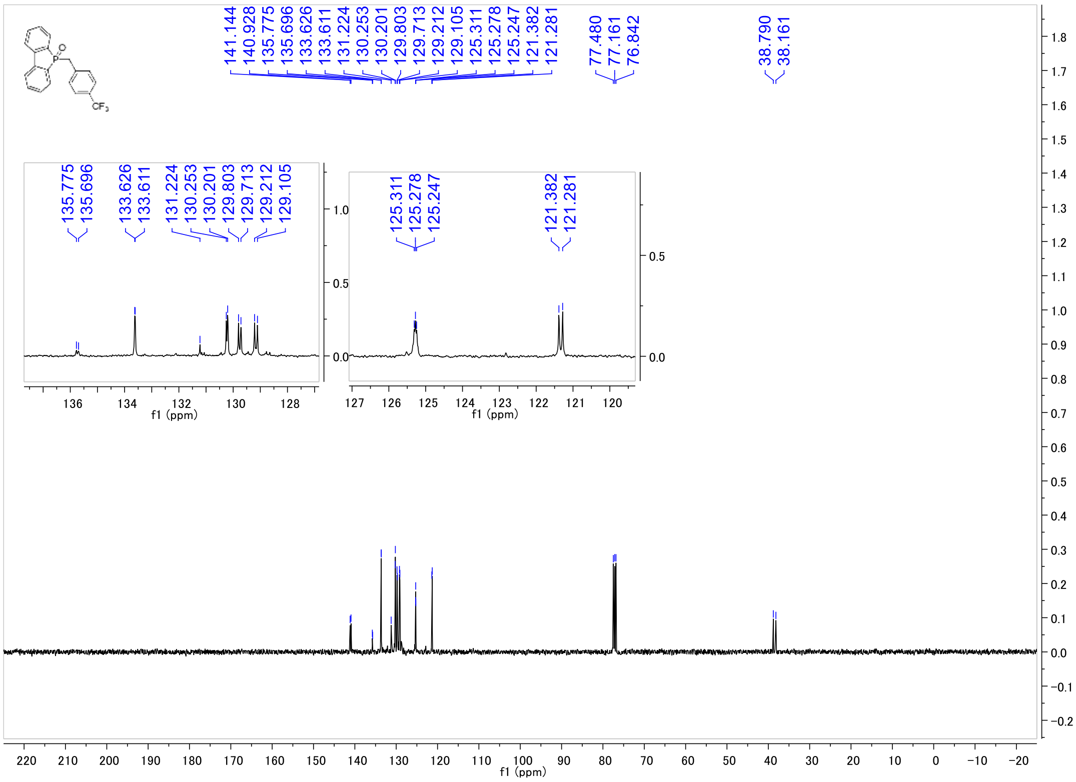

Supplementary Figure 40. ^{13}C NMR spectra of **3’f**

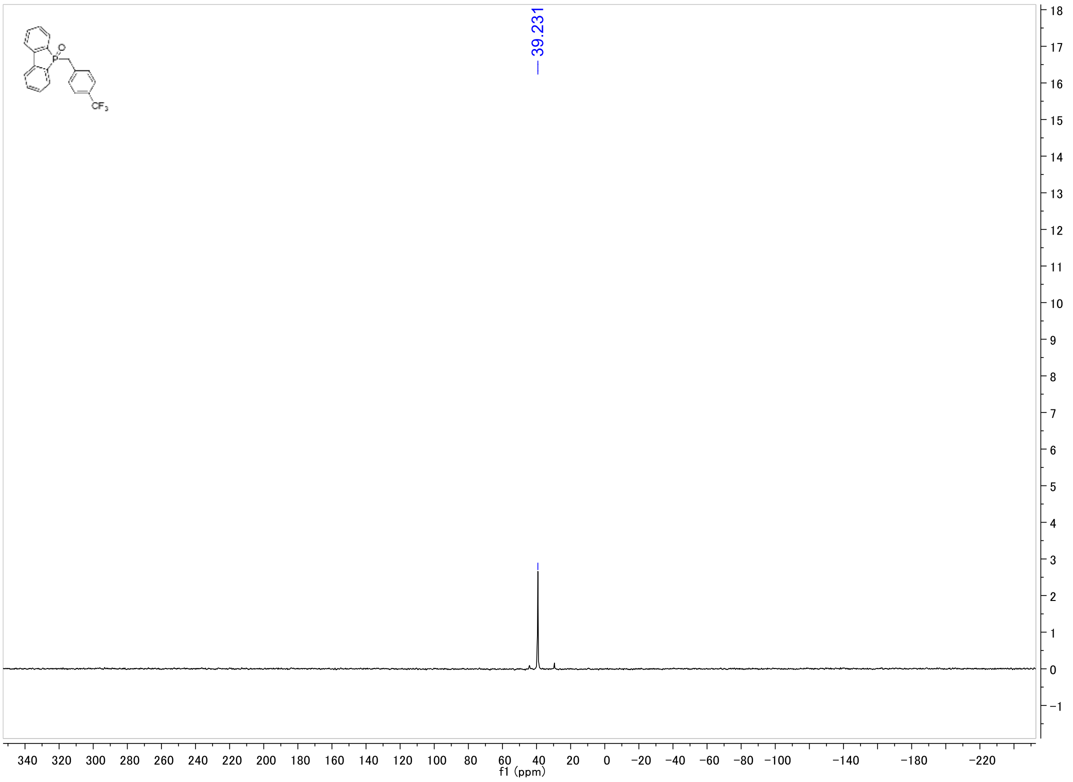

Supplementary Figure 41. ^{31}P NMR spectra of **3’f**

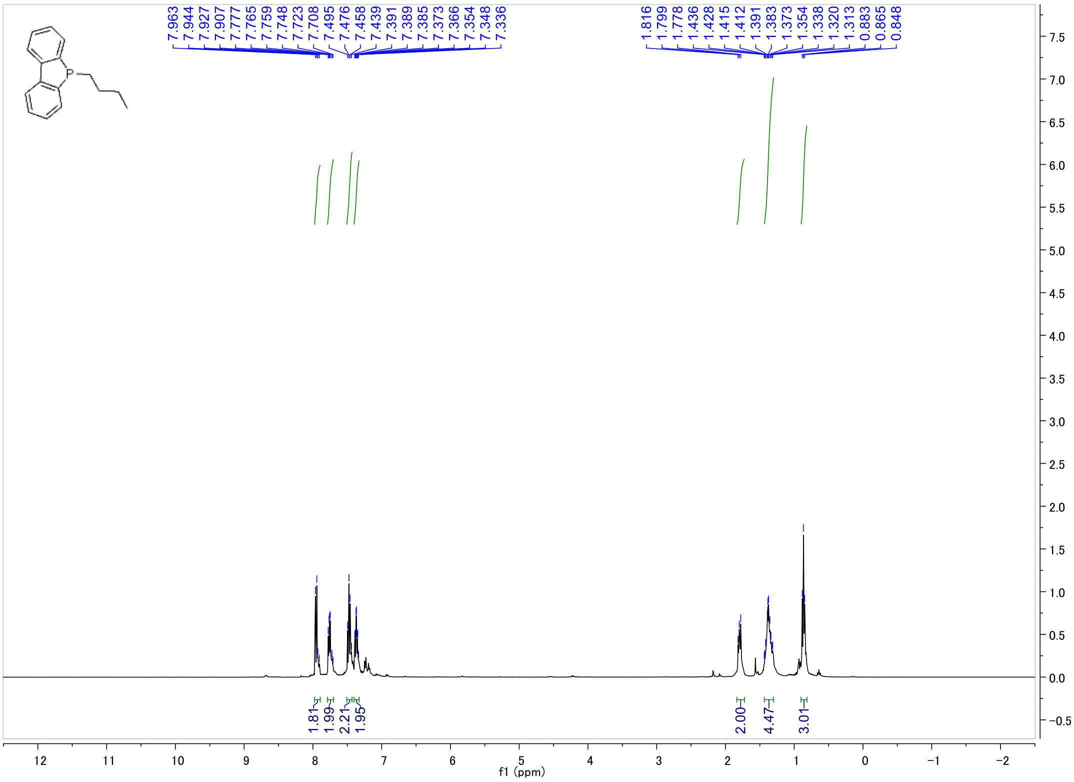

Supplementary Figure 42. ^{1}H NMR spectra of **4’a**

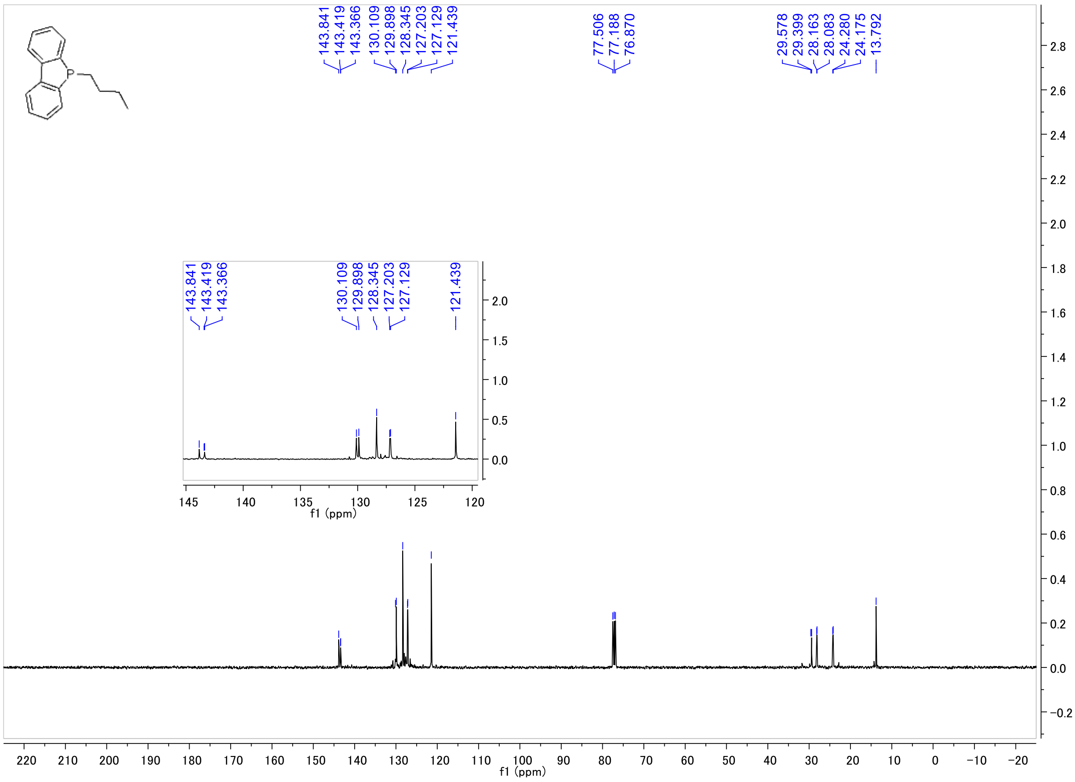

Supplementary Figure 43. ^{13}C NMR spectra of **4’a**

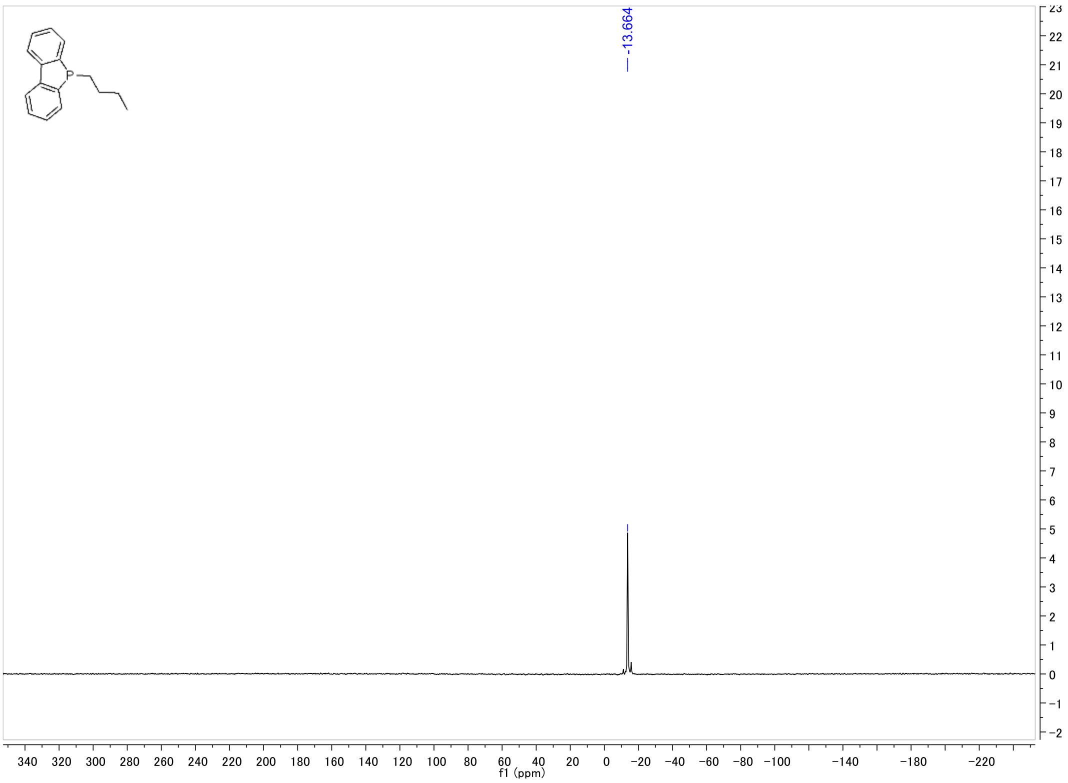

Supplementary Figure 44. ^{31}P NMR spectra of **4’a**

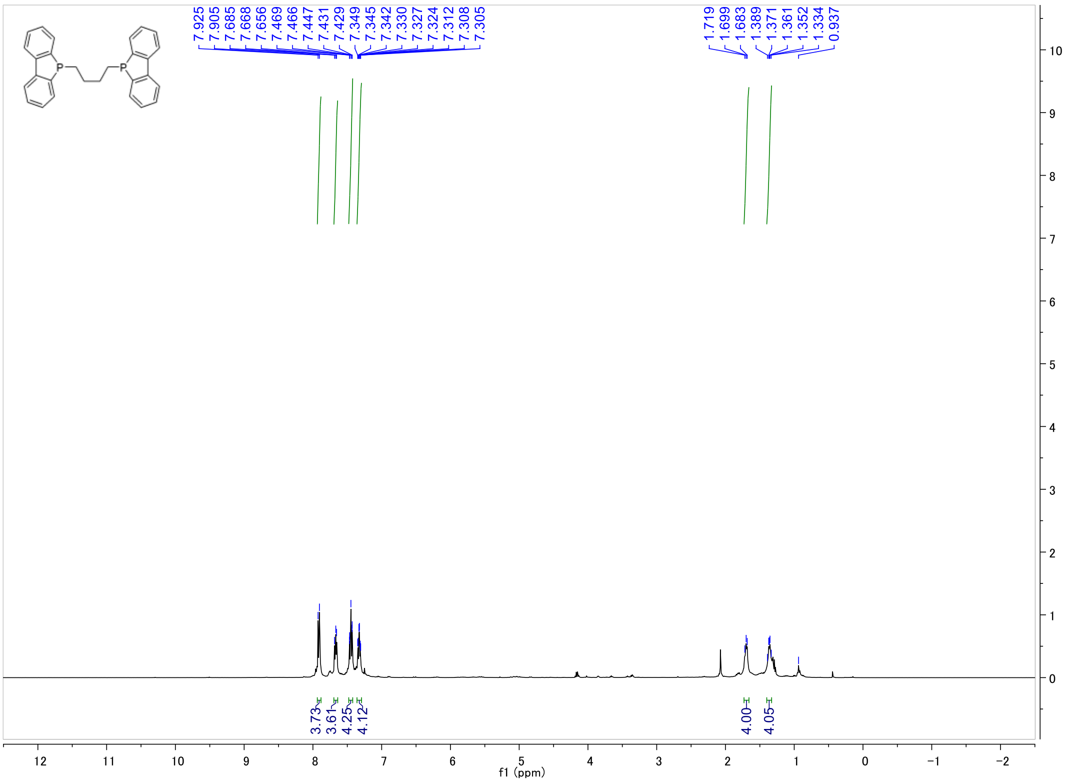

Supplementary Figure 45. ^{1}H NMR spectra of **4’b**

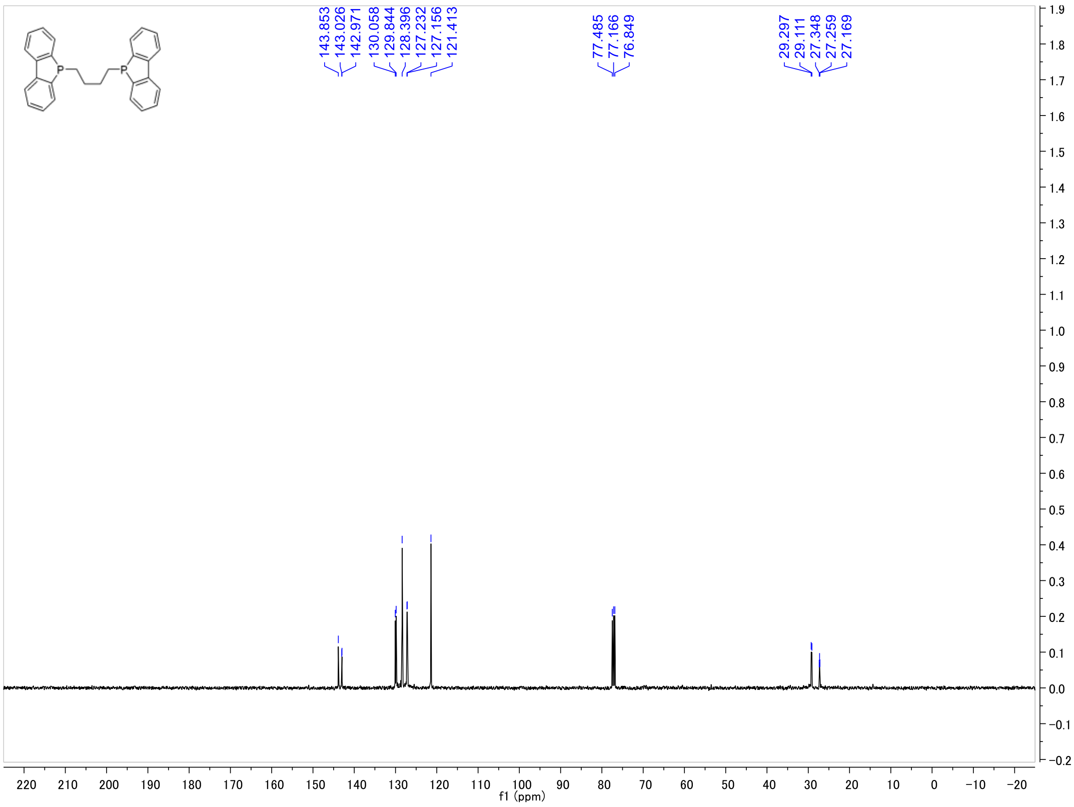

Supplementary Figure 46. ^{13}C NMR spectra of **4’b**

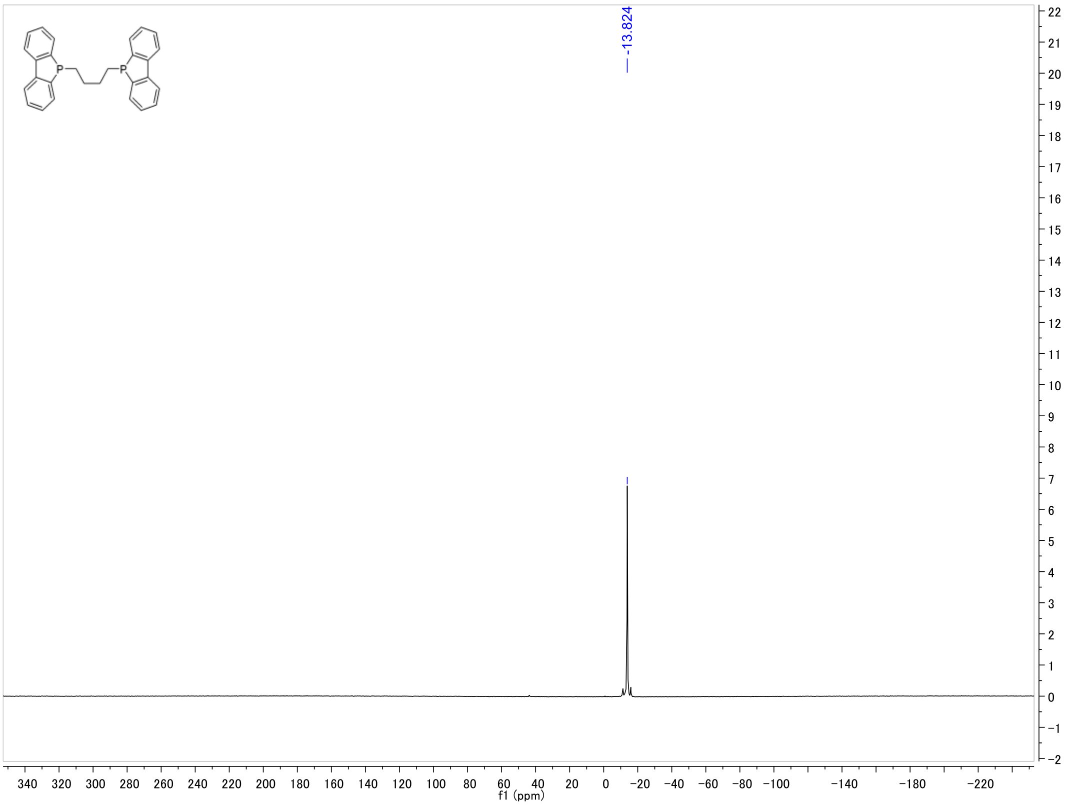

Supplementary Figure 47. ^{31}P NMR spectra of **4’b**

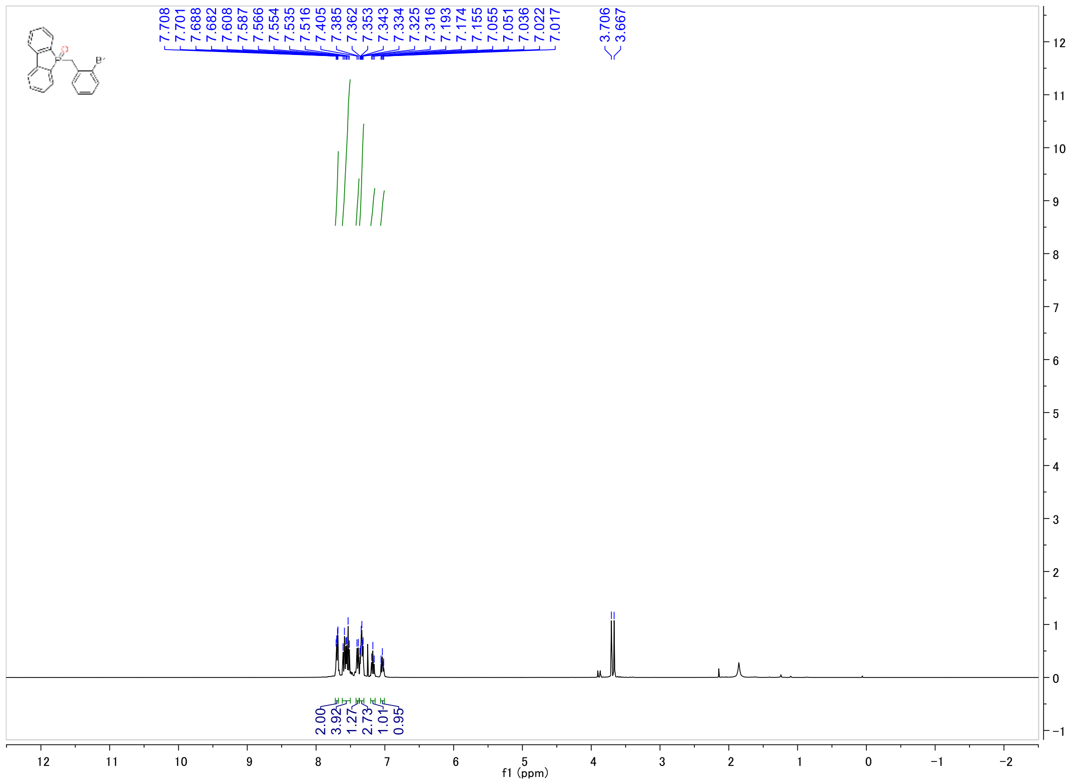

Supplementary Figure 48. ^{1}H NMR spectra of **4'c**

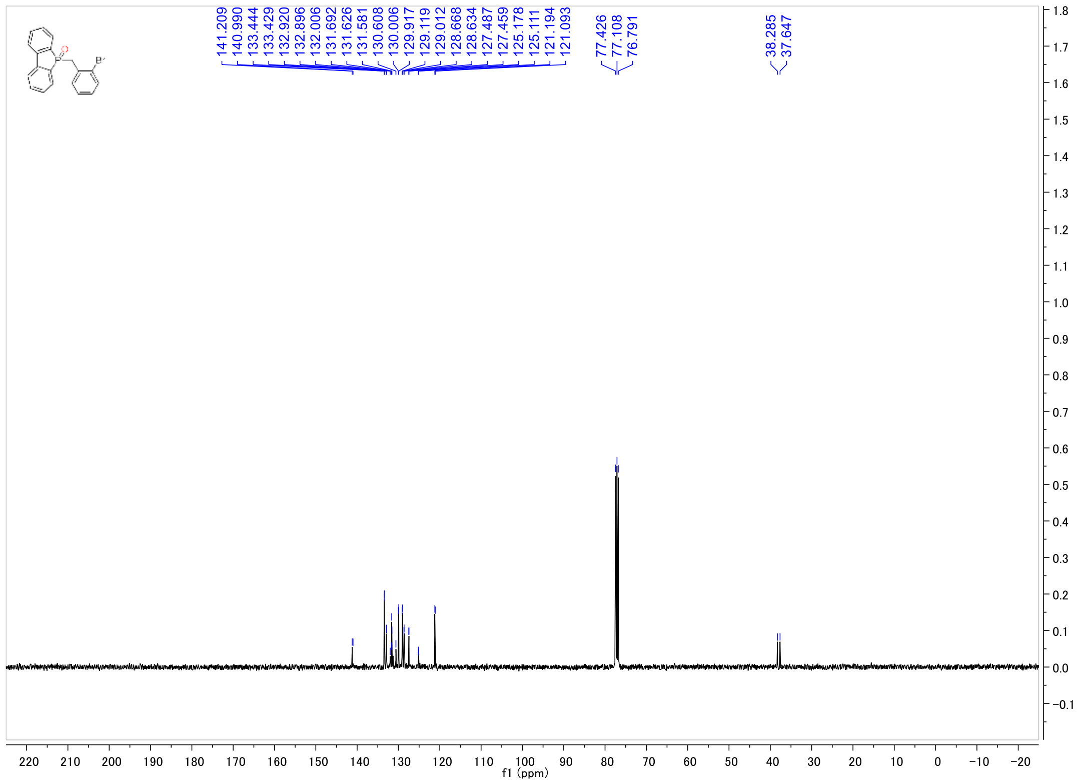

Supplementary Figure 49. ^{13}C NMR spectra of **4'c**

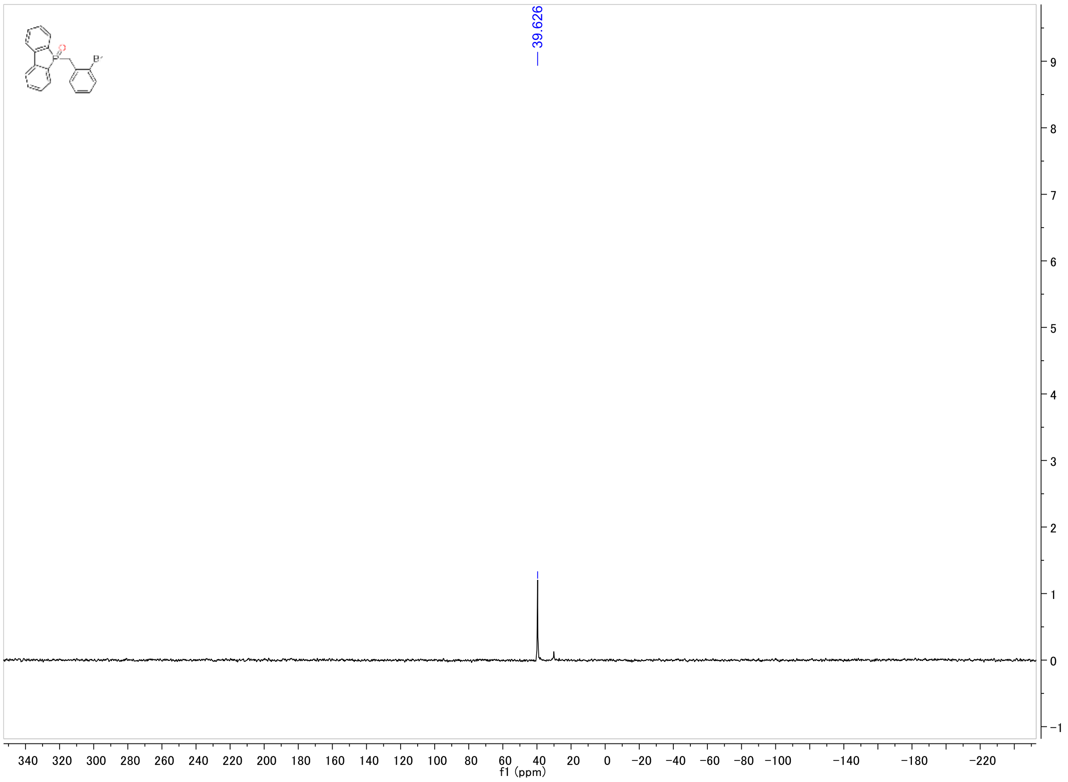

Supplementary Figure 50. ^{13}P NMR spectra of **4’c**
